# CD38 Is Robustly Induced in Human Macrophages and Monocytes in Inflammatory Conditions

**DOI:** 10.3389/fimmu.2018.01593

**Published:** 2018-07-10

**Authors:** Stephanie A. Amici, Nicholas A. Young, Janiret Narvaez-Miranda, Kyle A. Jablonski, Jesus Arcos, Lucia Rosas, Tracey L. Papenfuss, Jordi B. Torrelles, Wael N. Jarjour, Mireia Guerau-de-Arellano

**Affiliations:** ^1^Division of Medical Laboratory Science, School of Health and Rehabilitation Sciences, College of Medicine, Wexner Medical Center, The Ohio State University, Columbus, OH, United States; ^2^Division of Rheumatology and Immunology, Department of Internal Medicine, Wexner Medical Center, The Ohio State University, Columbus, OH, United States; ^3^Department of Microbial Infection and Immunity, The Ohio State University, Columbus, OH, United States; ^4^Department of Veterinary Biosciences, The Ohio State University, Columbus, OH, United States; ^5^Institute for Behavioral Medicine Research, The Ohio State University, Columbus, OH, United States; ^6^Department of Neuroscience, The Ohio State University, Columbus, OH, United States

**Keywords:** macrophage, monocyte, CD38, systemic lupus erythematosus, marker

## Abstract

Macrophages and their monocyte precursors mediate innate immune responses and can promote a spectrum of phenotypes from pro-inflammatory to pro-resolving. Currently, there are few markers that allow for robust dissection of macrophage phenotype. We recently identified CD38 as a marker of inflammatory macrophages in murine *in vitro* and *in vivo* models. However, it is unknown whether CD38 plays a similar marker and/or functional role in human macrophages and inflammatory diseases. Here, we establish that CD38 transcript and protein are robustly induced in human macrophages exposed to LPS (±IFN-γ) inflammatory stimuli, but not with the alternative stimulus, IL-4. Pharmacologic and/or genetic CD38 loss-of-function significantly reduced the secretion of inflammatory cytokines IL-6 and IL-12p40 and glycolytic activity in human primary macrophages. Finally, monocyte analyses in systemic lupus erythematosus patients revealed that, while all monocytes express CD38, high CD38 expression in the non-classical monocyte subpopulation is associated with disease. These data are consistent with an inflammatory marker role for CD38 in human macrophages and monocytes.

## Introduction

Chronic inflammatory diseases such as systemic lupus erythematosus (SLE) are commonly associated with functional deficits and a dramatically decreased quality of life. Innate immune responses mediated by macrophages and their monocyte precursors play a significant role as amplifiers and effectors of inflammation (1). Macrophages exposed to infectious and/or inflammatory stimuli adopt a phenotype characterized by high glycolytic activity and production of inflammatory cytokines and reactive oxygen species (ROS) [reviewed in Ref. (2)] aimed at pathogen destruction and immune effector recruitment. However, chronic activation of inflammatory macrophages may result in significant tissue and organ injury or dysfunction. Therefore, strategies that detect inflammatory macrophages may provide a means to diagnose inflammatory disease and/or follow treatment responsiveness.

CD38 was historically identified as a surface activation marker in T cells and later found to be expressed in additional immune and non-immune cell types, including macrophages (3). More recently, we found that CD38 is selectively upregulated in inflammatory murine bone marrow-derived macrophages (BMDM) (4). Other murine genes induced in inflammatory conditions are *N*-formyl peptide receptor (*Fpr2*) and G-protein receptor 18 (*Gpr18*), while *Egr2* is induced in M(IL-4) conditions (4). CD38 labels most inducible nitric oxide synthase (iNOS)^+^ macrophages stimulated in M(LPS + IFN-γ) conditions. By contrast, M(IL-4)-stimulated BMDM fail to express CD38 (4). *In vivo*, CD38^+/hi^ macrophages are also induced during infectious and sterile inflammatory processes such as murine models of *Listeria* infection (5, 6), LPS-induced sepsis (4), DSS-induced colitis (7), and focal ischemia (8). This pattern of expression is consistent with macrophage CD38 serving as an inflammatory marker in both *in vitro* and *in vivo* murine models.

Although the exact functional role of CD38 in macrophages is unclear, CD38 may regulate cellular processes *via* dual receptor and enzymatic activities. As a receptor, CD38 can bind platelet and endothelial cell adhesion molecule/CD31 to promote cell adhesion and transport across endothelial cell layers (9). However, CD38 was first described as an enzyme capable of catalyzing the conversion of nicotinamide-adenine dinucleotide (NAD) to cyclic ADP ribose (cADPR) *via* cyclase activity and cADPR to ADPR *via* hydrolase activity. In acidic conditions, such as in endocytic compartments, CD38 can additionally convert NAD phosphate (NADP) to nicotinic acid adenine dinucleotide phosphate (NAADP) (10). cADPR signals the ryanodine receptor to induce Ca^2+^ release from endoplasmic stores (11) and NAADP signals to Ca^2+^ stores in lysosomes (12). The Ca^2+^ signaling function of CD38 is evolutionarily conserved and is known to play crucial roles in infectious immunity, inflammation, and insulin signaling (3). Accordingly, CD38-deficient mice have increased susceptibility to infection as a consequence of reduced chemotactic activity and antigen presentation, modulation of bacterial uptake, and deficient T cell-dependent antibody and Th1 responses (5, 6, 13–18). Thus, murine animal models support a role for CD38 expression and activity in inflammatory disease processes.

CD38 is also associated with human inflammatory disease pathogenesis. In this context, both the presence and high percentage of CD38^+^ CD8 T cells are used as a biomarker to follow the progression of HIV infection into AIDS (19–21). Similarly, high percentages of CD38^+^ CD8 T cells are linked to anti-retroviral therapy unresponsiveness (19, 22). CD38 is also used clinically as a poor prognosis biomarker in small B cell chronic lymphocytic leukemia (23, 24). Moreover, CD38 expression is a prognostic biomarker in B cell acute lymphoblastic leukemia (25) and may play a role in multiple myeloma and acute promyelocytic leukemia (26). In addition, the anti-CD38 monoclonal antibodies daratumumab and isatuximab show therapeutic benefits in multiple myeloma (27–29) and are proposed as potential therapies for other conditions (30). However, while CD38 clearly plays a role in human disease and murine inflammation, little is known about its role in human inflammatory processes, particularly in macrophage-mediated innate immune responses.

Here, we ask whether CD38 can serve as an inflammatory marker in macrophages of human origin, similar to the murine system. In addition, we explore links between monocyte CD38 and disease activity in the human autoimmune-mediated inflammatory disease, SLE. Using both human monocytic cell line-derived and primary human monocyte-derived macrophages (MDMs), we report that surface CD38 is a selective marker of macrophages activated in M(LPS ± IFN-γ), but not M(IL-4) conditions. CD38 also contributes to maximal inflammatory cytokine secretion and glycolytic activity in human macrophages. Finally, we state that, while monocytes constitutively express CD38, high levels of CD38 expression in non-classical monocytes (NCMs) are linked to SLE disease and/or disease activity. Taken together, these data are consistent with an inflammatory marker role for macrophage/monocyte CD38 in human inflammatory processes.

## Materials and Methods

### Human Subjects IRBs and Ethics

To generate human MDM, peripheral blood mononuclear cells (PBMCs) were isolated from healthy donors (HD) by Ficoll gradient as previously described (31) under The Ohio State University Institutional Review Board (OSU-IRB) approval numbers 2008H0119, 2009H0132, and 2017H0040. Diagnosis and disease activity for SLE patients was made according to the revised criteria of the American College of Rheumatology for SLE (32). Patients with SLE and healthy volunteers were recruited for the study from OSU Wexner Medical Center clinics and local communities. All study participants were not currently taking hormonal medications and healthy age-/sex-matched samples were used in comparative analysis. Participation was through OSU-IRB approval 2009H0132. Informed consent was obtained from all human subject participants.

### Human Monocyte Cell Lines

THP-1 cells and U937 cells acquired from American Type Culture Collection (TIB-202 and CRL-10389, respectively) were cultured in complete RPMI, i.e., RPMI 1640 (Corning, Cellgro) supplemented with 10% fetal bovine serum (FBS) (Invitrogen), 1% penicillin/streptomycin (Cellgro), and 50 µM 2-mercaptoethanol (Sigma-Aldrich) (THP-1 cells) or with 10% FBS, 1% penicillin/streptomycin (U937 cells) at 37°C, 5% CO_2_. Cells were then plated at 5 × 10^5^ cells/ml, differentiated with 50 ng phorbol myristate acetate (Sigma-Aldrich) for 24 h, washed, and replated in fresh media for 48 h. To activate cells, medium was replaced with fresh media alone (M0), fresh media with 20 ng/ml IFN-γ (R&D) and 100 ng/ml LPS (Sigma-Aldrich) [M(LPS + IFN-γ)], or fresh media with 20 ng/ml IL-4 (R&D) [M(IL-4)]. After 24 h, cells were collected and analyzed for RNA or protein expression.

### Human MDM

To generate MDM, PBMCs were isolated from HD by Ficoll gradient and differentiated as previously described (31). Briefly, PBMCs were plated on Teflon wells in Phenol Red RPMI-1640 Media (Gibco) with 20% autologous serum at 2 × 10^6^ cells/ml for 5 days. On day 5, cells (mainly monocytes matured to become macrophages and surviving lymphocytes in suspension) in Teflon wells were collected and washed with cold RPMI. MDMs were further purified by establishing monolayers *via* adherence to tissue culture plates in the presence of RPMI containing 10% autologous or human AB serum at 5 × 10^6^ cells/well (12-well plate) and incubation for 2 h at 37°C. After washing out non-adherent lymphocytes, MDM monolayers were used for stimuli/inhibitor treatments or siRNA transfection.

For stimuli-only treatments, MDM monolayers were washed with PBS and treated with polarizing cytokines on day 6 in M0, M(LPS + IFN-γ), M(IL-4), M(LPS), M(IFN-γ), M(TNF-α), M(imiquimod, TLR7 agonist), M(R-848, TLR8 agonist), or M(IL-1β) conditions [LPS from *E. coli* O55:B5 at 100 ng/ml (Sigma-Aldrich), IFN-γ and IL-4 at 20 ng/ml each, TNF-α and IL-1β at 10 ng/ml (all from R&D Systems), imiquimod at 600 ng/ml, R-848 at 300 ng/ml (both from Enzo Life Sciences, Farmingdale, NY, USA)]. On day 7, supernatants were collected for ELISA while cells were washed with PBS and lysed in miRVana lysis buffer for RNA isolation or detached with accutase (BD Biosciences) or gentle scraping under cold conditions for flow cytometry.

For inhibitor treatments, MDM monolayers were treated on day 5 with vehicle, 15 µM rhein or 25 µM apigenin (both from Sigma-Aldrich and solubilized in DMSO). On day 6, cell-polarizing cytokines were added to the culture. Cells/supernatants were collected on day 7 for downstream analyses.

For siRNA transfections, MDM monolayers were transfected on day 5 with 100 µM CD38 silencer select siRNAs (Cat. No. 4392420, Assay IDs s2657 and s2659, 50 µM each, Life Technologies) or silencer select siRNA negative control (Cat. No. 4390844, Life Technologies) using TransIT-TKO transfection reagent (Mirus Bio LLC, Madison, WI, USA) in RPMI. On day 6, transfection medium was replaced with RPMI with 10% autologous or human AB serum and cell-polarizing cytokines in M0 or M(LPS + IFN-γ) conditions were added to the culture. Cells/supernatants were collected on day 7 for downstream analyses.

### SLE Patient/Healthy Control Samples

Biological samples were collected either from venous blood draws directly into Ficoll-containing CPT tubes [BD Vacutainer^®^ CPT™ Mononuclear Cell Preparation Tube—Sodium Heparin (16 mm × 125 mm/8 ml)] or into heparinized tubes. PBMCs were subsequently isolated from whole blood according to previously described methods using Ficoll-Paque centrifugation (GE Healthcare, Uppsala, Sweden) as described in Ref. (33), or following CPT™ tube’s manufacturer’s protocol.

### RNA Isolation

To examine RNA expression, macrophage RNA was isolated using the miRVana kit (Life Technologies) according to the manufacturer’s specifications. RNA quality/concentration was quantified using a Nanodrop spectrophotometer (Thermo Fisher Scientific). Samples were stored at −80°C until analysis.

### Real-Time PCR

Macrophage mRNA gene expression was determined using Taqman quantitative Real-Time PCR on cDNA templates. cDNA was generated from 500 ng RNA/sample using oligo(dT)_12–18_ primers and Superscript III (Life Technologies), according to the manufacturer’s instructions. Product was amplified with commercially available Taqman primers (Life Technologies) and probe sets with Taqman Mastermix (Roche) on an Applied Biosystems 7300 Real-Time PCR or StepOnePlus thermocycler. The assay ID numbers for ABI sets were the following: *CD38*—Hs-1120071_m1, *FPR2*—Hs02759175_s1, *GPR18*—Hs01921463_s1, *EGR2*—Hs00166165_m1, and *c-MYC*—Hs00153408_m1. Expression of target genes was normalized to *βACTIN*/*HPRT* as a loading control. Real-Time PCR data were analyzed using the comparative Ct (ΔΔCt) method.

### Flow Cytometry

Peripheral blood mononuclear cells or MDMs were resuspended in FACS buffer and blocked with anti-human FcR antibody (CD16/CD32, Miltenyi) for 15 min at 4°C in FACS buffer (PBS with 2% FBS and 1 mM EDTA). Cells were then surface stained with antibodies against CD3 (clone UCHT1, FITC or BV-510, BioLegend), CD4 (clone RPA-T4, APC-Cy7, BioLegend), CD8 (clone SK1, APC, BioLegend), CD11b (clone ICRF44, PE-Cy7, BioLegend), CD19 (clone HIB19, BV-510, BioLegend), CD20 (clone 2H7, PerCP-Cy5.5, BioLegend), CD38 (clone HIT2, PE, BioLegend), CD45 (clone H130, PB, BioLegend), CD56 (clone HCD56, BV-510, BioLegend), CD66b (clone G10F5, FITC, BioLegend), CD14 (clone 61D3, PerCP-Cy5.5 or FITC, eBioscience), CD16 (clone eBioCB16, APC, eBioscience), CD40 (clone 5C3, e450, eBioscience), FPR2 (clone 304405, APC, R&D Systems), or isotype control for 15 min at 4°C. Cells were washed three times with FACS buffer, fixed in 2% paraformaldehyde, washed three times with FACS buffer, resuspended in FACS buffer, and run on a BD FACSCanto II or BD LSRII Flow Cytometer (BD, NJ, USA) within 24 h of processing. Data were analyzed with Flow Jo (Tree Star, OR, USA).

### Cytokine ELISA

Cytokines were detected post-stimulation using a sandwich ELISA. All reagents were purchased from BioLegend: Human IL-6 (Capture: 501101, Detection: 501201, Standard: 570809), human IL-12p40 (Capture: 501702, Detection: 508801, Standard: 572109). ELISAs were performed as previously described (34).

### Cell Death Assay

Cell death in drug-treated or transfected MDM was measured *via* release of intracellular lactate dehydrogenase (LDH) (Pierce™ LDH Cytotoxicity Assay Kit, Cat No 88953, Thermo Fisher Scientific). Supernatants were collected from established MDM monolayers just prior to cell processing on day 7 and processed according to the manufacturer’s instructions. Absorbance was measured at 490 and 680 nm. LDH activity was determined by subtracting the 680 nm absorbance value (background) from the 490 nm absorbance value.

### Glycolysis Assay

Extracellular l-lactate, the end product of glycolysis, was measured in either drug-treated or transfected MDM using the Glycolysis Cell-Based Assay Kit (Cat. No. 600450, Cayman Chemical, Ann Arbor, MI, USA). Supernatants were collected from established MDM monolayers just prior to cell processing on day 7 and processed according to the manufacturer’s instructions. Absorbance was measured at 490 nm.

### Statistics

Statistical significance was determined by unpaired or paired *t* tests (two-tailed, equal SD) or analysis of variance (ANOVA) with *p* values adjusted for multiple comparisons using Sidak’s, Dunnett’s, or Tukey’s multiple comparisons test. *p* < 0.05 was considered to be significant. Correlation analyses were performed using linear regression with either Pearson’s correlation analysis if the samples passed the D’Agostino and Pearson normality test or Spearman’s correlation analysis if they did not. All analyses were completed using GraphPad Prism 6.0 or 7.0 software.

## Results

### CD38 Is Upregulated in Human M(LPS + IFN-γ) Monocytic Cell Line-Derived Macrophages

**Figure 1:**
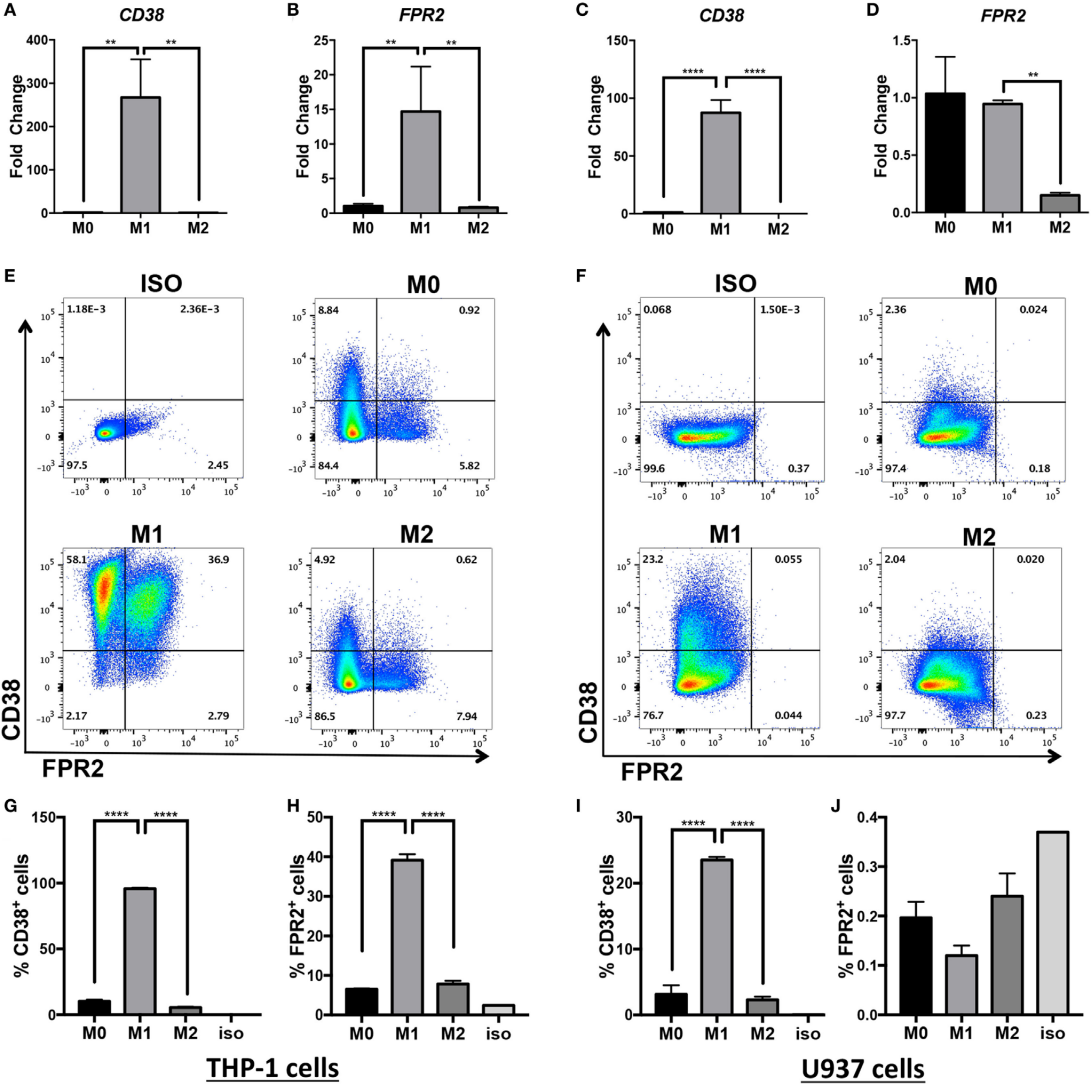
CD38 mRNA and protein expression is increased in human monocytic cell lines differentiated into M(LPS + IFN-γ) macrophages. Expression of *CD38* and *FPR2* mRNA in THP-1 **(A,B)** and U937-derived macrophages **(C,D)** in unstimulated (M0), M(LPS + IFN-γ)-stimulated (labeled M1 throughout figure), or M(IL-4)-stimulated (labeled M2 throughout figure) macrophages was measured by real-time PCR and expressed as mean relative expression ± SD (*n* = 3 biological replicates with two technical replicates per sample). Gene expression is expressed as fold change ± SD relative to M0 condition. One-way analysis of variance with *p* values adjusted for multiple comparisons using Sidak’s multiple comparisons test compare M(LPS + IFN-γ) vs. M0 and M(LPS + IFN-γ) vs. M(IL-4). **(E,F)** Flow cytometry staining of surface FPR2 on *x*-axis, CD38 on *y*-axis in THP-1 **(E)** and U937 **(F)** cells. Flow plots correspond to total cells. Data shown are representative of *n* = 3 biological replicates. **(G–J)** Quantification of CD38^+^ cells and FPR2^+^ cells in THP-1 **(G,H)** or U937 cells **(I,J)** are expressed as percent of positive cells ± SD (*n* = 3 biological replicates); ISO, isotype control; **p < 0.01, ****p < 0.0001.

**Figure 2:**
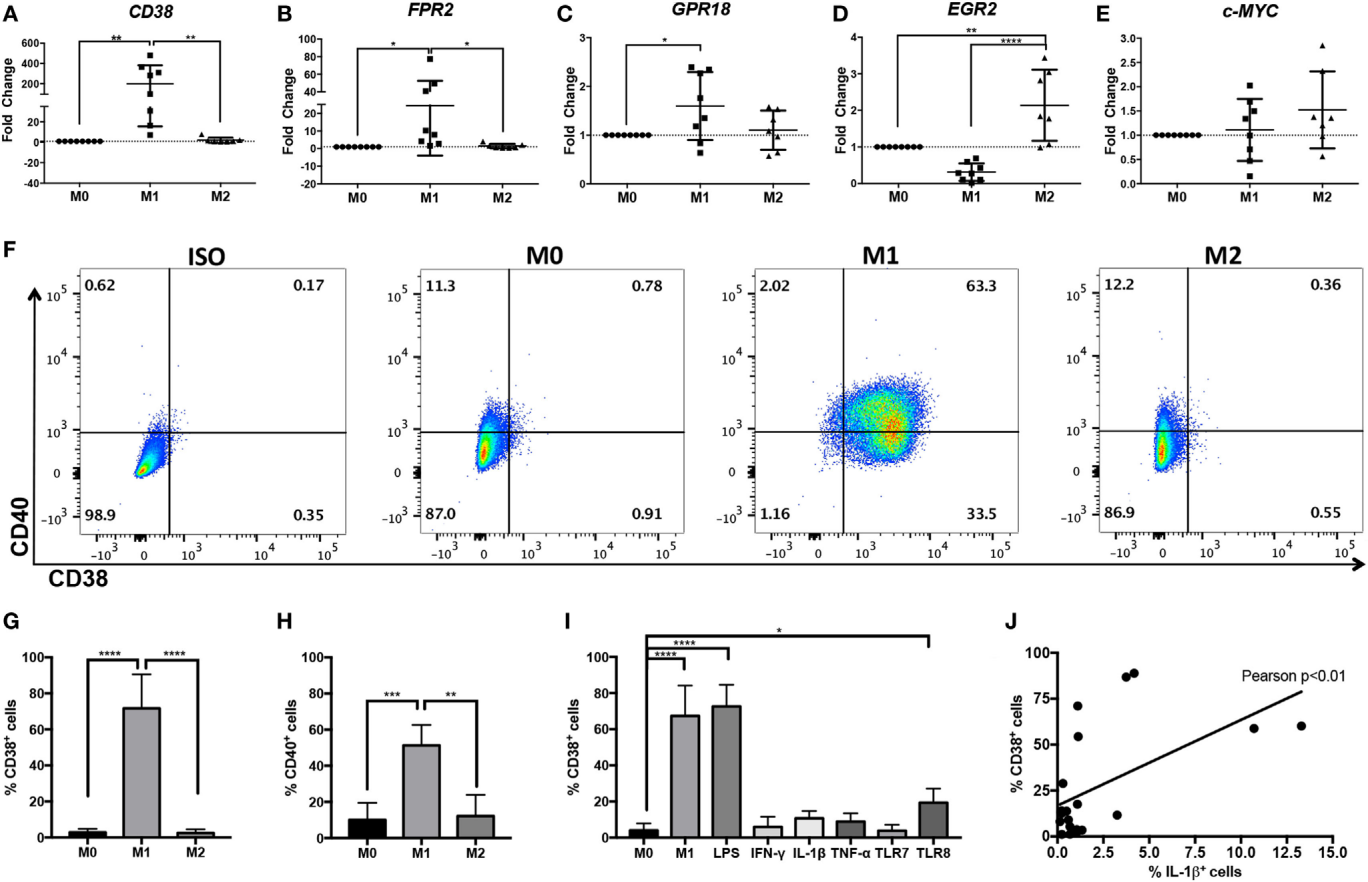
Increased CD38 expression in human M(LPS + IFN-γ) monocyte-derived macrophages (MDMs). Expression of human *CD38* **(A)**, *FPR2* **(B)**, *GPR18* **(C)**, *EGR2* **(D)**, and *c-MYC* **(E)** genes in human MDMs in unstimulated (M0), M(LPS + IFN-γ) (labeled M1 throughout figure), or M(IL-4) (labeled M2 throughout figure) human MDMs, as measured by real-time PCR. Gene expression level is expressed as fold change ± SD relative to M0 condition (*n* = 7–8 independent samples, each generated from different donors; each independent sample value corresponds to the average of two technical replicates). One-way analysis of variance (ANOVA) with *p* values adjusted for multiple comparisons using Sidak’s multiple comparisons test, *p < 0.05, **p < 0.01, ****p < 0.0001. **(F)** Flow cytometry staining of surface CD38 and CD40 at 24 h post-differentiation into M0, M(LPS + IFN-γ), or M(IL-4) macrophages. Flow plots correspond to CD11b^+^ cells. Data shown are representative of *n* = 4 independent samples generated from different individual donors; ISO, isotype control. **(G,H)** Quantification of CD38^+^ cells **(G)** or CD40^+^ cells **(H)** is expressed as percent of cells ± SD (*n* = 4 independent samples generated from different individual donors). One-way ANOVA with *p* values adjusted for multiple comparisons using Sidak’s multiple comparisons test, **p < 0.01, ***p < 0.001, ****p < 0.0001. **(I)** Quantification of flow cytometry staining of surface CD38^+^ cells at 24 h post-differentiation into M0, M(LPS + IFN-γ), M(LPS), M(IFN-γ), M(IL-1β), M(TNF-α), M(TLR7), or M(TLR8) macrophages is expressed as percent of cells ± SD (*n* = 3 samples from MDM generated in three independent experiments from three distinct donors, with two technical replicates per sample). One-way ANOVA with *p* values adjusted for multiple comparisons using Dunnett’s multiple comparisons test, ****p < 0.0001. **(J)** Pearson’s correlation analysis between CD38^+^ cells and IL-1β^+^ cells. The straight line represents linear regression calculation, *r* = 0.5512, Pearson’s **p < 0.01.

Inflammatory macrophages contribute to a wide range of inflammatory diseases, so markers that allow robust and consistent detection of murine and human inflammatory macrophages are necessary. Since human inflammatory monocytes/macrophages inconsistently express the classical iNOS marker (35, 36), current available markers are limited to CD40 detection or intracellular detection of IL-6, IL-12, and IL-1β cytokines requiring *in vitro* culture. Our laboratory previously discovered that CD38, an ectoenzyme and surface receptor, serves as a robust *in vitro* and *in vivo* marker of inflammatory murine macrophages (4). *Fpr2* transcripts are also upregulated in BMDM activated in M(LPS + IFN-γ) conditions (4). To determine whether CD38 and FPR2 are similarly induced in human inflammatory macrophages, we activated differentiated THP-1 and U937 macrophages in M0, M(LPS + IFN-γ) (labeled M1 in Figures 1 and 2), or M(IL-4) (labeled M2 in Figures 1 and 2) conditions. *CD38* and *FPR2* gene expression was evaluated by real-time PCR 24 h post-activation (Figures 1A–D). *CD38* was robustly upregulated in M(LPS + IFN-γ) vs. M0 and M(IL-4) conditions in both THP-1 (Figure 1A, ~270-fold increase) and U937 cells (Figure 1C, ~90-fold). To a lesser extent, *FPR2* mRNA expression was also increased in THP-1 cells activated in M(LPS + IFN-γ) conditions (Figure 1B, ~15-fold increase). By contrast, there was no *FPR2* induction in M(LPS + IFN-γ) U937 macrophages compared with M0 but, consistent with THP-1 cells, there was a significant decrease in cells cultured in M(IL-4) vs. M(LPS + IFN-γ) conditions (Figure 1D).

To confirm that *CD38* and *FPR2* gene induction was present at the protein level, we examined surface CD38 and FPR2 expression by flow cytometry (Figures 1E–J). Indeed, similar to its transcript expression pattern, THP-1 and U937 macrophages induced surface CD38 expression after LPS + IFN-γ activation. Approximately 96% of THP-1 cells (Figures 1E,G) stimulated in M(LPS + IFN-γ) conditions expressed CD38, compared with ~10% in M0 and ~5% in M(IL-4) cells (Figures 1E,G). FPR2 was also significantly and selectively induced in ~40% of THP-1 M(LPS + IFN-γ) vs. ~8% of M(IL-4) cells (Figures 1E,H), with double-positive CD38^+^FPR2^+^ cells only observable in M(LPS + IFN-γ) conditions (Figure 1E). The induction of CD38 was more muted in U937 cells, with ~23% of M(LPS + IFN-γ) cells expressing CD38 vs. ~3% CD38^+^ cells in M0 and ~2% in M(IL-4) conditions (Figures 1F,I). By contrast, FPR2 protein expression was not detectable in U937 cells (Figures 1F,J), consistent with gene expression data (Figure 1D). Overall, these data indicate that while FPR2 induction is cell-line dependent, CD38 serves as a surrogate marker of human M(LPS + IFN-γ) activation in two different monocytic cell line-derived macrophages.

### CD38 Is Induced in M(LPS + IFN-γ) but Not M(IL-4) Human MDM

While THP-1 and U937 macrophages are often used to model human macrophages, primary human MDM provide a model that more closely reflects *ex vivo* macrophages. Therefore, we explored whether CD38 and FPR2 were also induced in primary human MDM activated with LPS + IFN-γ. We also explored the homologs of murine M(LPS + IFN-γ) marker *Gpr18* and M(IL-4) markers *Egr2* and *c-Myc*, which we had previously identified in BMDM murine macrophages (4). Primary human MDMs were activated with LPS + IFN-γ or IL-4 and analyzed by real-time PCR or flow cytometry. The results largely recapitulated our data generated from murine macrophage and human monocytic cell lines; *CD38* (Figure 2A) and *FPR2* (Figure 2B) transcripts were significantly and robustly induced (mean fold change: 198.4 for *CD38* and 24.4 for *FPR2*) in M(LPS + IFN-γ, labeled M1)-, but not M(IL-4, labeled M2)-stimulated MDM. Human *GPR18* gene transcripts were also significantly, albeit only mildly, upregulated (mean fold change: 1.6) in M(LPS + IFN-γ) MDM (Figure 2C). *EGR2* was significantly (mean fold change: 2.1), upregulated in M(IL-4) conditions and decreased in M(LPS + IFN-γ) conditions (mean fold change: 0.3), relative to M0 macrophages (Figure 2D). By contrast, *c-MYC* expression was not consistently induced in M(IL-4) macrophages (Figure 2E).

CD38 flow cytometry analysis showed that, while the majority (three quarters) of M(LPS + IFN-γ) macrophages were CD38^+^, fewer than 3% of M0 and M(IL-4) macrophages stained for CD38 protein (Figures 2F,G). These results indicate that CD38 is highly restricted to inflammatory M(LPS + IFN-γ) macrophages. To compare CD38 to the current human M(LPS + IFN-γ) marker CD40, we evaluated its expression by flow cytometry and observed that CD40 only labeled half of the M(LPS + IFN-γ) population and also labeled up to 12% of M0 or M(IL-4) macrophages (Figure 2H) (37). These results indicate that CD38 labels more M(LPS + IFN-γ) macrophages than CD40 and fewer non-inflammatory M(0) and M(IL-4) macrophages than CD40. To determine which stimuli can induce CD38, we evaluated CD38 expression after exposure to various individual stimuli. We found that LPS alone resulted in levels of CD38 induction similar to those of M(LPS + IFN-γ) and that IFN-γ alone did not induce CD38 (Figure 2I). Among other cytokines tested, neither TNF-α nor IL-1β significantly induced CD38 (Figure 2I). Similar results were obtained with the TLR7 agonist imiquimod, while the TLR8 agonist R-848 mildly induced CD38 (Figure 2I). CD38 expression correlated with the secretion of the inflammatory cytokine IL-1β, further supporting the link between CD38 and inflammatory macrophages (Figure 2J). In summary, these data indicate that CD38 is a robust and selective marker of human inflammatory M(LPS ± IFN-γ) macrophages.

### CD38 Promotes Inflammatory Cytokine Secretion in Human Macrophages

**Figure 3:**
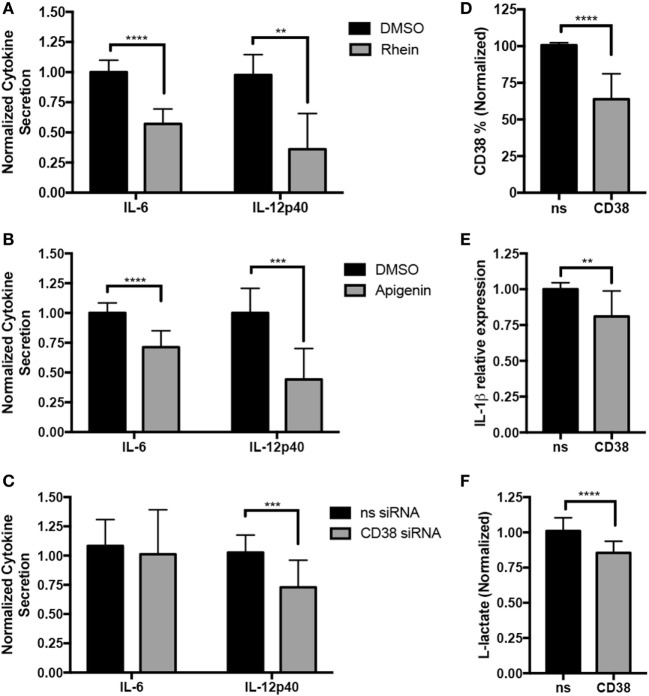
CD38 promotes inflammatory cytokine secretion in human macrophages. Human monocyte-derived macrophages (MDMs) were treated with 15 µM rhein or DMSO control **(A)**, 25 µM apigenin or DMSO **(B)**, or transfected with 100 µM CD38 siRNA cocktail or siRNA control **(C)** on day 5. On day 6, they were activated with LPS + IFN-γ for an additional 24 h prior to analysis. **(A–C)** IL-6 and IL-12p40 secretion was analyzed by ELISA from MDM supernatants. Graphs pool normalized (relative to corresponding experiment vehicle control or nonsense siRNA) data from three **(A)**, five **(B)**, or six **(C)** independent experiments/donors, with at least two technical replicates for each sample. Data are expressed as mean cytokine secretion ± SD relative to vehicle condition. **(D)** Quantification of CD38^+^ cells analyzed by flow cytometry from MDM transfected with control or CD38 siRNAs for data shown in panel **(C)** is expressed as percent of cells ± SD, *n* = 6 independent experiments/donors and two technical replicates per sample. **(E)** Quantification of IL-1β^+^ MDMs analyzed by flow cytometry for data shown in panel **(C)** is expressed as percent of cells ± SD, *n* = 6 independent experiments/donors and two technical replicates per sample. **(F)** l-Lactate assays run using supernatants from panel **(C)**, *n* = 5 independent experiments/donors, with at least three technical replicates per sample. All data were analyzed by unpaired *t* tests. **p < 0.01, ***p < 0.001, ****p < 0.0001.

The increased expression of CD38 in inflammatory macrophages raises the question of whether CD38 promotes inflammatory responses. To answer this question, loss-of-function experiments were performed. Since no exclusive CD38 inhibitors exist, we used rhein (38) and apigenin (39), two CD38 inhibitors that, respectively, originate from the structurally distinct anthraquinone and flavonoid families. This strategy was expected to reduce the likelihood of shared rhein and apigenin effects being mediated by any possible off-target effects (as off-target effects are expected to differ between rhein and apigenin). We treated MDM from HD with rhein or apigenin and subsequently differentiated in M(LPS + IFN-γ) conditions in the presence/absence of inhibitors (Figure 3). Rhein significantly suppressed IL-6 and IL-12p40 secretion by M(LPS + IFN-γ) macrophages, as determined by ELISA (Figure 3A, 43 and 64% decreases, respectively, vs. DMSO, results normalized and combined from three experiments, *n* = 5–10). Single representative experiments with absolute values for experiments are shown in Figures S1A–C in Supplementary Material. The unrelated CD38 inhibitor apigenin similarly suppressed IL-6 and IL-12p40 secretion (Figure 3B, 34 and 51% decreases, respectively, vs. DMSO, results normalized and combined from five experiments, *n* = 10). Cytokine suppressive effects were not due to increased cell death, as no differences in cell viability between the control DMSO and treated conditions were present (Figure S2 in Supplementary Material). Since both rhein and apigenin can target other proteins (40, 41), we sought further confirmation *via* genetic method that CD38 was responsible for the observed effects (Figure 3C). We induced CD38 knockdown in MDM with two CD38-targeting siRNAs and then differentiated to M(LPS + IFN-γ) conditions. Although CD38 knockdown reduced CD38^+^ cells by only ~35% (Figure 3D) and did not significantly reduce IL-6, significantly suppressive effects on IL-12p40 cytokine secretion (27% decrease vs. DMSO, results normalized and combined from six experiments, *n* = 14) were observed (Figure 3C). In addition, we explored inflammatory IL-1β cytokine secretion and glycolysis metabolic activity, *via* secretion of the glycolysis end-product l-lactate, both of which increase in inflammatory macrophages (2). We observed a mild but significant decrease in both IL-1β (19% decrease) (Figure 3E) and lactate secretion (15% decrease) (Figure 3F) in CD38 siRNA conditions. Cytokine and lactate suppressive effects were not due to increased cell death, as no differences in cell viability between the control and CD38 siRNA were observed (Figure S2 in Supplementary Material). Therefore, data are consistent with CD38 activity or expression contributing to maximal IL-12 and IL-1β inflammatory cytokine secretion and glycolytic activity in human inflammatory macrophages.

### Enhanced CD38 Expression in NCMs From Active SLE Patients

**Figure 4:**
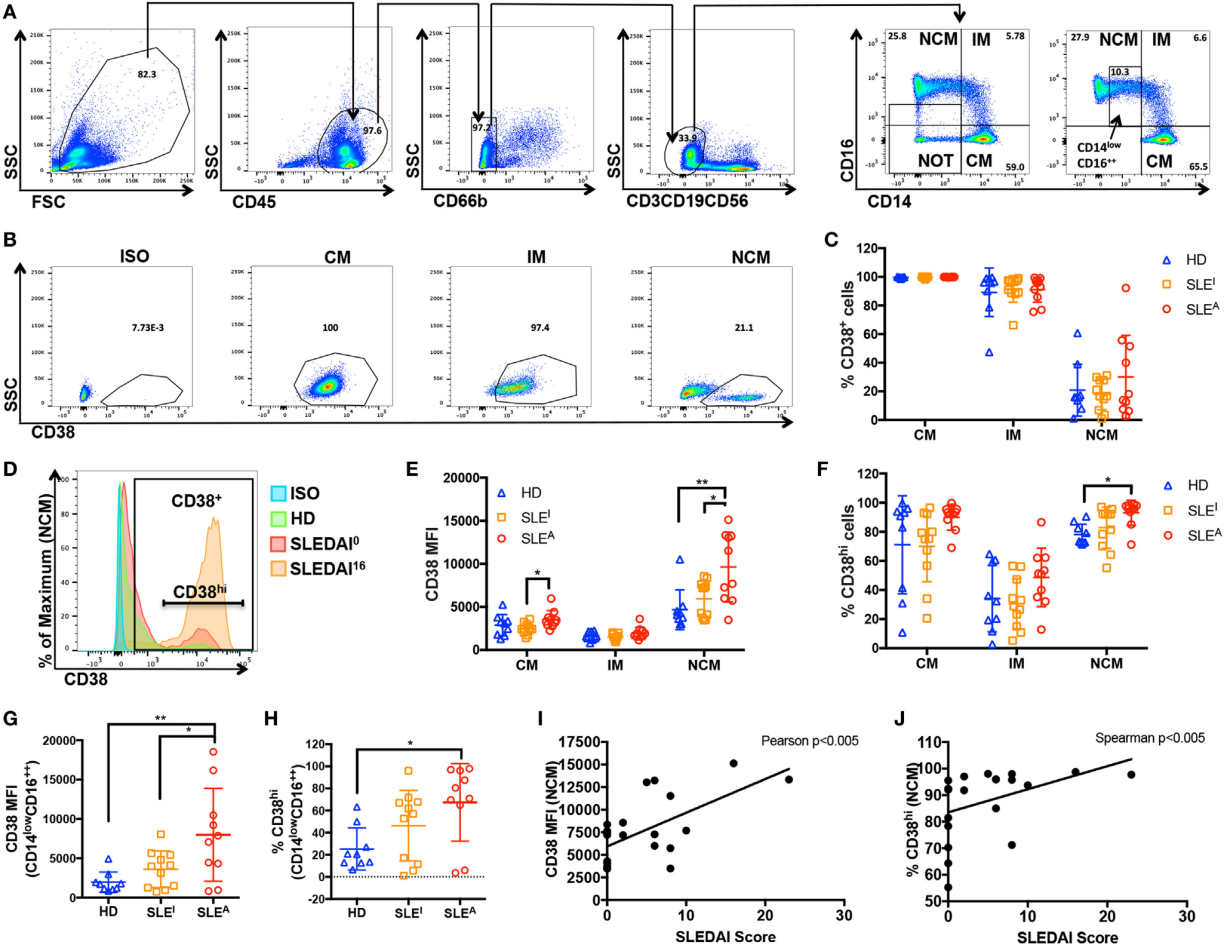
CD38 mean fluorescence intensity (MFI) in non-classical monocytes (NCMs) is elevated in active systemic lupus erythematosus (SLE). **(A)** Gating strategy: peripheral blood mononuclear cells isolated from healthy donors (HD) or SLE patients (panel 1) were gated for CD45^+^ cells (panel 2) prior to gating out CD66b^−^ cells (panel 3) and CD3^−^CD19^−^CD56^−^ (panel 4) cells. The remaining population was analyzed with CD14 and CD16 after removing cells that are neither CD14^+^ nor CD16^+^ (NOT gate) (panel 5) to identify classical (CD14^++^CD16^−^) (CM), intermediate (CD14^+^CD16^+^) (IM) and non-classical (CD14^+^CD16^+^) (NCM) monocytes (panel 6). A stricter gate that excluded CD14^−^ cells was also used for some analyses (box in panel 6 labeled CD14^low^CD16^++^). **(B)** Example of how the relative percentage of CM, IM, and NCM among monocytes was calculated and how CM, IM, and NCM populations were analyzed for CD38 expression. ISO, isotype. **(C)** Percent of CD38^+^ cells within the CM, IM and NCM subsets in HD, inactive (SLE^I^ = SLEDAI 0), or active (SLE^A^ = SLEDAI > 4) patients. **(D)** A histogram of CD38 expression in NCM indicating how the CD38^hi^ subset was defined. A histogram from each group is overlaid, including an SLE^I^ patient with an SLE Disease Activity Index (SLEDAI) of 0 and an SLE^A^ patient with an SLEDAI of 16. **(E)** CD38 MFI within CD38^+^ CM, IM, and NCM populations of HD, SLE^I^, and SLE^A^ patients. **(F)** Percent of CD38^hi^ cells within CM, IM, and NCM subsets in HD, SLE^I^, or SLE^A^ patients. **(G)** CD38 MFI within CD38^+^ CD14^low^CD16^++^ population in HD, SLE^I^, or SLE^A^ patients. **(H)** Percent of CD38^hi^ cells within CD14^low^CD16^++^ population in HD, SLE^I^, or SLE^A^ patients. **(C,E–H)** Quantification of CD38^+^ cells **(C)** and CD38^hi^ cells **(F,H)** is expressed as percent of positive cells ± SD. Quantification of CD38 MFI **(E,G)** is expressed as MFI of positive cells ± SD. *n* = 9 donors for HD, *n* = 11 patients for SLE^I^, and *n* = 10 patients for SLE^A^. Each sample was run in duplicate, and duplicate values were averaged prior to analysis. One-way analysis of variance with *p* values adjusted for multiple comparisons using Tukey’s multiple comparisons test, *p < 0.05, **p < 0.01. **(I,J)** Correlation analyses of SLEDAI score with NCM CD38 MFI **(I)** or percent of CD38^hi^ NCM **(J)** in active and inactive SLE patients. **(I)** CD38 MFI has a Gaussian distribution as determined by D’Agostino and Pearson normality test, so Pearson correlation analysis was performed, *r* = 0.6265, **p < 0.005. **(J)** Percent of CD38^hi^ cells does not have a Gaussian distribution as determined by D’Agostino and Pearson normality test, so Spearman correlation analysis was performed, *r* = 0.6085, **p < 0.005.

The association between exposure to an inflammatory stimulus such as LPS ± IFN-γ and expression of CD38 raises the question of whether myeloid CD38 plays a role in human inflammatory diseases. SLE is a systemic inflammatory autoimmune disease characterized by autoantibody generation and immune complex-mediated tissue damage. Disease activity can vary widely and can be assessed clinically using the standardized SLE Disease Activity Index (SLEDAI) scoring system, which is based on clinical signs (such as fever, seizures, and muscle weakness) and laboratory values (including blood cell counts or anti-DNA antibody titers) (42). We analyzed CD38 expression in monocytes from HD, inactive SLE (SLE^I^) patients with an SLEDAI score of 0–2, and active SLE (SLE^A^) patients with SLEDAI scores over 4 (see Table 1 for patient demographics). There were no statistically significant differences in age, gender, or race among the three groups (Table 1). To exclude potential contaminating granulocytes, NK cells, T cells, and B cells from the CD45^+^ population, we gated out cells positive for CD66b, CD56, CD3, and CD19, respectively. We then examined CD14 and CD16 expression levels to differentiate various types of monocytes, the precursors of tissue macrophages (Figure 4A, top row). CD14^++^CD16^−^ cells correspond to classical monocytes (CM), while CD14^++^CD16^+^ correspond to intermediate monocytes (IM) and CD14^+^CD16^++^ to NCMs (Figure 4A, panels 5–6), as previously described (43, 44). The inflammatory potential of each of these populations is controversial. While initial studies suggested that CM and IM were inflammatory and NCM were regulatory, attempts to functionally characterize the inflammatory potential of monocyte subsets have often been contradictory (45–55). Contrary to what was originally thought, several studies have now shown that NCM produce higher levels of inflammatory cytokines in response to inflammatory stimuli than CM (50, 54, 55). To identify links between SLE disease and monocyte CD38, we analyzed CD38 expression within CM, IM, and NCM. In contrast to the lack of CD38 expression on unstimulated macrophages, the majority of unstimulated CM and IM expressed CD38 on the surface while NCM had two distinct CD38 populations (Figures 4B,C). CD38 monocytic expression was not surprising as CD38 plays an important role in extravasation of blood monocytes into tissues (3). The % CD38^+^ cells did not change with SLE diagnosis or activity (Figure 4C). We reasoned that CD38 level, rather than mere presence, may be associated with inflammatory disease activity. To address this question, we compared the mean fluorescence intensity (MFI) of CD38^+^ cells and the percentage of CD38^hi^ cells (as defined in Figure 4D) in HD and SLE patients (Figures 4E,F). The MFI of CD38^+^ NCM, a measure of CD38 protein load, was significantly higher in active SLE^A^ patients (MFI = 9,646) than in inactive SLE^I^ patients (MFI = 5,949) or HD (MFI = 4,667). It is possible that CD38 expression levels are associated with age, gender, or race. Instead, we found no significant correlation between age and NCM CD38 MFI (Figure S3A in Supplementary Material) or significant differences between female and male (Figure S3B in Supplementary Material) or Caucasian and African-American NCM CD38 MFI (Figure S3C in Supplementary Material). The CD38 MFI of CM was also significantly increased in active SLE^A^ (MFI = 3,543) vs. inactive SLE^I^ patients (MFI = 2,888) (Figure 4E). The increase in NCM MFI was also reflected by a small but significant increase in percent of CD38^hi^ NCM in active SLE patients compared with HD (93 vs. 78%) (Figure 4F). Although we found no significant correlation between age and NCM CD38^hi^% (Figure S3D in Supplementary Material), there was a non-significant trend toward increased NCM CD38^hi^% in females vs. males (Figure S3E in Supplementary Material), indicating that CD38^hi^% may be subject to gender variation. We found no significant difference between races (Figure S3F in Supplementary Material). To rule out that any non-monocyte CD14^−^ cells were responsible for increased CD38 in NCM analyses, we re-analyzed data using a more restrictive gating (named CD14^low^CD16^++^ in Figure 4A, panel 6). This analysis, shown in Figures 4G,H, exactly reproduced the larger NCM gate data in Figures 4D,E. To further ascertain whether NCM CD38 MFI or % CD38^hi^ cells is linked to SLE disease activity, we performed correlation analyses in the SLE patient population, identifying significant positive correlations between NCM CD38 MFI and % CD38^hi^ and SLEDAI score (Figures 4I,J). Overall, these data indicate that CD38 is a marker of steady-state monocyte populations and that SLE disease activity is associated with CD38 MFI in a subset of NCMs, which have inflammatory potential.

**Table 1:** Donor demographics.

Group	Healthy	SLE^I^	SLE^A^
*N*	9	11	10
Average age (years ± SD)^a^	39.9 ± 14.1	41.5 ± 16.8	32.5 ± 7.3
SLE Disease Activity Index (avg ± SD)	N/A	0.4 ± 0.8	9.6 ± 5.7
Gender (F/M)	6/3	9/2	10/0
Race (C/A/U)	5/3/1	5/4/2	6/3/1
**Therapies**			
Azathioprine	0	2	4
Clobetasol 0.05% foam	0	1	0
Cyclosporine	0	0	1
Doxepin HCL	0	1	0
Hydroxychloroquine	0	9	7
Leflunomide	0	0	1
Meloxicam	0	1	0
Mycophenolate mofetil	0	3	1
Prednisone	0	2	7
Valaciclovir	0	0	1

*SLE^I^, inactive systemic lupus erythematosus; SLE^A^, active systemic lupus erythematosus; F, female; M, male; C, Caucasian; A, African-American; U, unknown or other race*.

*^a^Age analysis: one-way analysis of variance with p values adjusted for multiple comparisons using Tukey’s multiple comparisons test; HD vs. SLE^I^; HD vs. SLE^A^; SLE^I^ vs. SLE^A^; p = ns for all comparisons*.

## Discussion

Here, we report the novel finding that surface CD38 protein is robustly and selectively induced in M(LPS + IFN-γ) human macrophages. We also observe selective induction of the *FPR2* and *GPR18* genes by LPS + IFN-γ and of *EGR2* gene by IL-4 in primary human macrophages. We found that inhibition of CD38 activity or expression in primary inflammatory macrophages interfered with full inflammatory IL-12 and IL-1β cytokine production and glycolytic activity. In the chronic inflammatory disease SLE, we observed higher CD38 expression in NCM from active vs. inactive SLE patients or healthy controls, suggesting an association of CD38 to inflammatory disease activity. We also observed significant correlations between NCM CD38 MFI or CD38^hi^% and SLEDAI disease scores. Overall, these findings are consistent with the idea that CD38 plays a conserved role in inflammatory macrophages.

Since its early identification in human leukocytes, CD38 has served as a practical surface T and B cell differentiation and activation marker [reviewed in Ref. (3)]. The identification in 2015 of CD38 as a murine inflammatory macrophage marker that effectively dissects inflammatory from alternatively activated macrophages (4) raised the possibility that CD38 could also serve as an inflammatory marker in human macrophages. Here, we find that LPS + IFN-γ treatment induces CD38 at the transcriptional and protein level in multiple human macrophage models ranging from monocyte-derived cell lines to primary MDM. These results are consistent with CD38 representing a conserved response to inflammatory stimulation. Here, we induced inflammatory macrophages with a combination of LPS + IFN-γ, but other inflammatory stimuli also promote CD38. IFN-γ alone induces CD38 in human monocytes (56), although we did not observe this effect in human macrophages (Figure 2I). Similarly, TNF-α (57) and TLR9 agonists (58) have also been reported to induce CD38 in smooth muscle cells and leukemic cells, respectively, but neither TNF-α, IL-1β nor TLR7 agonist imiquimod induced CD38 in human macrophages (Figure 2I). The only individual stimuli that induced CD38 were LPS and the TLR-8 agonist R-848, albeit to a much lesser extent (Figure 2I). CD38 expression is positively correlated with IL-1β expression and was present in a much higher proportion of M(LPS + IFN-γ) macrophages than the current M(LPS + IFN-γ) human macrophage marker CD40. In addition, it was practically absent from M0 and M(IL-4) macrophages, providing improved resolution over CD40. In summary, CD38 is a highly and consistently induced inflammatory marker conserved from mouse to human macrophage responses. Therefore, CD38 may serve as a marker to evaluate the effects of small molecules on inflammatory macrophage phenotype modulation or monitor inflammatory disease status.

CD38 is a complex surface molecule that can play multiple roles, each one of which may have different functional effects. CD38 can act as an ectoenzyme on the cell surface, depleting extracellular NAD or NADP, and producing mediators cADPR, ADPR, and NAADP that promote intracellular Ca^2+^ increase, proliferation, and insulin secretion (59). On the other hand, CD38 can act as a receptor with ability to bind CD31 on endothelial and other cells, thereby promoting endothelial adhesion as well as cell activation and proliferation (3, 30). Here, we attempted to explore the functional role of CD38 in macrophages using CD38 inhibitors rhein and apigenin. Both these treatments suppressed IL-6 and IL-12, consistent with CD38 promoting inflammatory cytokine production. A caveat to this interpretation is that, while both rhein and apigenin inhibit CD38 activity, they can also impact other pathways. For example, besides targeting CD38, rhein also inhibits NF-κB and P2X7 (60) and IKKβ (61) and suppresses IL-6 and NO (61). Apigenin also affects other macrophage signaling pathways, including NF-κB. Apigenin can inhibit LPS-induced IL-6 and IL-1β production and caspase-1 and ERK1/2 inhibition has been observed with apigenin (62). Apigenin also reduces expression of miR-155 (63), a miRNA that promotes inflammatory macrophage phenotype (64) and activates PPARγ, promoting M(IL-4) phenotypes (65). Although it is unclear whether CD38 inhibition is occurring upstream or in parallel to these effects, it is possible that the inflammatory cytokine secretion effects are mediated by other pathways. To overcome these limitations, we attempted to knockdown CD38. Although only a suboptimal knockdown (35%) of CD38 was achieved, knockdown was mirrored by similar decreases in IL-12/IL-1β secretion and glycolytic activity. By contrast, no significant IL-6 decrease was observed, suggesting that rhein and apigenin’s effects on IL-6 are mediated by pathways other than CD38.

Based on these intriguing results in macrophages, it will be interesting to further investigate the role of decreased vs. increased CD38 expression in monocyte function and activity. For example, baseline expression of CD38 in monocytes is expected to mediate endothelial cell adhesion, which is necessary for monocyte extravasation into tissues. However, high CD38 expression, as in the case of CM and NCM from active SLE patients, may promote inflammatory cytokine production or drive monocyte-to-macrophage differentiation. This hypothesis will be tested in future experiments. From studies in dendritic cells, neutrophils, and monocytes, CD38 is known to be essential for trafficking and chemotaxis to sites of infection (13, 14, 16, 66, 67). More recently, CD38 has been found to be necessary for resistance to *Listeria* infections *via* NAD depletion and actin cytoskeleton modulation (18). In this model, a significant reduction in *Il1b, Il6, Il12b*, and *TNFa* inflammatory cytokine transcripts was observed in CD38 KO macrophages, while *Nos2* and *Cox2* transcripts were unchanged (18). We now show that CD38 expression and activity promotes inflammatory cytokine production and glycolytic activity in human macrophages. Since NO is not produced by human inflammatory macrophages, it was not evaluated. However, it remains to be determined if CD38 also plays a role in generation of ROS.

Our results show increased CD38 expression (MFI and CD38^hi^%) in NCM from SLE^A^ patients compared with healthy controls. In addition, NCM MFI from SLE^A^ patients was also significantly higher than that of SLE^I^ patients, suggesting that the extent of CD38 expression is linked to disease activity in SLE. Indeed, correlation analyses between NCM CD38^hi^% or MFI and SLEDAI score confirmed this prediction. Besides inflammatory monocytes/macrophages, activated T cells and B cells, plasma cells, and NK cells also express high levels of CD38 (3, 30). In contrast to previous reports (68–70), we observed no significant changes in CD38 in SLE patients’ T cells and B cells (Figure S4 in Supplementary Material). The reason for this discrepancy is unclear but sample size is a likely factor as we observed a trend toward increases in both B cells and T cells. Our results are consistent with a role for CD38 in SLE, as previously suggested *via* links between CD38 and the Fas^lpr^ murine models of SLE (71) and CD38 SNPs and severe discoid rashes in SLE patients (72). Since our data suggest that CD38 expression and activity are associated with SLE disease diagnosis and severity, future studies could further explore the marker and functional role of CD38 in larger cohorts, as well as whether genetic variation at this locus is linked to inflammatory pathology.

Therapeutic targeting of CD38 is currently used for multiple myeloma and other hematologic malignancies (30) and has also been proposed for SLE and other antibody-mediated autoimmune diseases (73). Among several anti-CD38 antibody therapies in development, daratumumab is the most advanced. Daratumumab binds an epitope of CD38, inhibiting its cyclase activity and efficiently promoting CD38^+^ cell lysis *in vivo* (30, 74). Consequently, anti-CD38 antibody therapy has been proposed as a method to deplete pathogenic autoantibody-producing long-lived plasma cells in SLE (73). Our results raise the possibility that, in addition to effects on plasma cells, anti-CD38 therapy may modulate inflammatory CD38^+^ monocytes and macrophages. However, it is difficult to predict the effects of CD38 antibodies, as they may differentially impact the receptor function and the various enzymatic activities of CD38. For example, while daratumumab inhibits CD38 cyclase activity, it in turn enhances hydrolase enzymatic activity (30). It will be important to evaluate in detail what these effects are and how monocyte/macrophage CD38 blockade modulates anti-cancer/anti-SLE therapeutic effects. Consistent with therapeutic effects of anti-CD38 therapy in SLE, anti-thymocyte antibody treatments that include CD38 antibodies suppress SLE and other autoimmune diseases (73). It is also interesting to note that SLE patients with anti-CD38 autoantibodies have lower levels of clinical activity and decreased titers of pathogenic anti-dsDNA antibodies (69). Perhaps the greatest challenge of targeting CD38 therapeutically stems from its broad expression pattern on different cell types and its multifunctional nature, as it acts both as a receptor and has various ectoenzyme activities. For instance, although CD38 deficiency impairs immune responses and disease pathogenesis in murine arthritis and asthma models (59, 75), it can instead exacerbate murine models of lupus and diabetes (76, 77). Since CD38 deficiency did not enhance pathogenic autoimmune responses, exacerbation effects appear to be due to non-immune effects on target tissue (59, 76). Overall, these results highlight the need for better understanding the functional role of CD38 and its receptor and ectoenzyme activity in individual cell types, as well as of specific cellular targeting.

In conclusion, CD38 induction in inflammatory macrophages is conserved from mouse to humans. In addition, high CD38 expression in NCM is associated with active SLE disease and CD38 activity contributes to inflammatory cytokine release. These novel findings suggest that CD38 may be a useful marker of inflammatory macrophage/monocyte-mediated disease and warrant additional clinical and mechanistic studies to fully define its diagnostic and/or therapeutic targeting potential.

## Ethics Statement

This study was carried out in accordance with the recommendations of Ohio State University Office of Responsible Research Practices Institutional Review Board with written informed consent from all subjects. All subjects gave written informed consent in accordance with the Declaration of Helsinki. The protocol was approved by The Ohio State University Institutional Review Board.

## Author Contributions

Conceived and designed the experiments: SA, NY, TP, JT, WJ, and MG. Performed the experiments: SA, NY, JN-M, KJ, JA, and LR. Analyzed the data: SA, NY, JN-M, KJ, JA, LR, and MG. Wrote the paper: SA, NY, KJ, TP, JT, WJ, and MG.

## Conflict of Interest Statement

The authors declare that the research was conducted in the absence of any commercial or financial relationships that could be construed as a potential conflict of interest.

## Supplementary Material

Figure S1 Representative graphs with absolute values from individual experiments for Figure 3. Human monocyte-derived macrophages (MDMs) were treated with 15 µM rhein or DMSO control **(A)**, 25 µM apigenin or DMSO **(B)**, or transfected with 100 µM CD38 siRNA cocktail or nonsense siRNA control **(C)** on day 5. On day 6, they were activated with LPS + IFN-γ for an additional 24 h prior to analysis. **(A–C)** IL-6 and IL-12p40 secretion was analyzed by ELISA from MDM supernatants. Graphs show data (relative to corresponding experiment vehicle control or nonsense siRNA) from a single experiment, one to two biological replicates with two technical replicates per sample. All data were analyzed by unpaired *t* tests. **p* < 0.05, ***p* < 0.01, ****p* < 0.001.

Figure S2 Lactate dehydrogenase (LDH) remains unchanged in treatment groups compared with corresponding experiment vehicle control or nonsense siRNA. **(A–C)** Quantification of LDH activity from monocyte-derived macrophage supernatants of 15 µM rhein-treated **(A)**, 25 µM apigenin-treated **(B)**, or CD38 siRNA-transfected **(C)** conditions compared with control conditions shown in Figure 3. All graphs represent pooled data from three to five independent experiments with one to two biological replicates per experiment and two to three technical replicates per sample. All data were analyzed by unpaired *t* tests.

Figure S3 There are no significant associations between CD38 expression and age, gender, or race. CD38 mean fluorescence intensity (MFI) **(A–C)** and % CD38hi cells **(D–F)** from healthy donors (HD) were analyzed to determine if age, gender or race affected CD38 protein load or surface expression. **(A,D)** Correlation analyses of SLE Disease Activity Index score with non-classical monocytes (NCM) CD38 MFI **(A)** or % CD38^hi^ NCM **(D)** in HD. **(A)** CD38 MFI did not pass D’Agostino and Pearson normality test, so Spearman correlation analysis was performed, *r* = −0.1506, *p* = ns, *n* = 9. **(D)** Percent of CD38^hi^ NCM has Gaussian distribution as determined by D’Agostino and Pearson normality test, so Pearson correlation analysis was performed, *r* = −0.2617, *p* = ns, *n* = 9. **(B,C,E,F)** CD38 MFI **(B,C)** and % CD38^hi^ cells **(E,F)** in NCM from HD were analyzed by gender **(B,D)**, *n* = 3 males (M) and 6 females (F); or race **(C,F)**, *n* = 5 Caucasians; *n* = 3 African-Americans, *n* = 1 unknown. Data analyzed by unpaired *t* tests, *p* = ns.

Figure S4 CD38^+^ T cell and B cell percentages in patients with SLE. **(A)** Gating strategy: peripheral blood mononuclear cells isolated from healthy donors or SLE patients (panel 1) were gated for CD45^+^ cells (panel 2), then CD20^+^ or CD3^+^ cells (panel 3), and subsequently CD38^+^ cells **(B,C)**. **(B,C)** Quantification of CD45^+^CD20^+^CD38^+^ **(B)** or CD45^+^CD3^+^CD38^+^ **(C)** cells are expressed as percent of positive cells ± SD, *n* = 9 for HD, *n* = 11 for SLE^I^, *n* = 10 for SLE^A^. One-way analysis of variance with Tukey’s multiple comparisons test.

## References

[1] Moulton VR Suarez-Fueyo A Meidan E Li H Mizui M Tsokos GC. Pathogenesis of human systemic lupus erythematosus: a cellular perspective. Trends Mol Med (2017) 23:615–35. 10.1016/j.molmed.2017.05.006 28623084 PMC5650102

[2] Amici SA Dong J Guerau-de-Arellano M. Molecular mechanisms modulating the phenotype of macrophages and microglia. Front Immunol (2017) 8:1520. 10.3389/fimmu.2017.01520 29176977 PMC5686097

[3] Malavasi F Deaglio S Funaro A Ferrero E Horenstein AL Ortolan E Evolution and function of the ADP ribosyl cyclase/CD38 gene family in physiology and pathology. Physiol Rev (2008) 88:841–86. 10.1152/physrev.00035.2007 18626062

[4] Jablonski KA Amici SA Webb LM de Dios Ruiz-Rosado J Popovich PG Partida-Sánchez S Novel markers to delineate murine M1 and M2 macrophages. PLoS One (2015) 10:e0145342. 10.1371/journal.pone.0145342 26699615 PMC4689374

[5] Lischke T Heesch K Schumacher V Schneider M Haag F Koch-Nolte F CD38 controls the innate immune response against *Listeria monocytogenes*. Infect Immun (2013) 81:4091–9. 10.1128/IAI.00340-13 23980105 PMC3811837

[6] Botta D Rivero-Nava L Lund F The NAD glycohydrolase CD38 regulates macrophage effector function and defense against *Listeria monocytogenes*. (INC7P.409). J Immunol (2014) 192(1 Suppl) 186.10.

[7] Schneider M Schumacher V Lischke T Lücke K Meyer-Schwesinger C Velden J CD38 is expressed on inflammatory cells of the intestine and promotes intestinal inflammation. PLoS One (2015) 10:e0126007. 10.1371/journal.pone.0126007 25938500 PMC4418770

[8] Choe CU Lardong K Gelderblom M Ludewig P Leypoldt F Koch-Nolte F CD38 exacerbates focal cytokine production, postischemic inflammation and brain injury after focal cerebral ischemia. PLoS One (2011) 6:e19046. 10.1371/journal.pone.0019046 21625615 PMC3097994

[9] Deaglio S Morra M Mallone R Ausiello CM Prager E Garbarino G Human CD38 (ADP-ribosyl cyclase) is a counter-receptor of CD31, an Ig superfamily member. J Immunol (1998) 160:395–402. 9551996

[10] Lee JW Choi CH Choi JJ Park YA Kim SJ Hwang SY Altered microRNA expression in cervical carcinomas. Clin Cancer Res (2008) 14:2535–42. 10.1158/1078-0432.CCR-07-1231 18451214

[11] Lee HC. Structure and enzymatic functions of human CD38. Mol Med (2006) 12:317–23. 10.2119/2006-00086.Lee 17380198 PMC1829193

[12] Churchill GC Okada Y Thomas JM Genazzani AA Patel S Galione A. NAADP mobilizes Ca(2+) from reserve granules, lysosome-related organelles, in sea urchin eggs. Cell (2002) 111:703–8. 10.1016/S0092-8674(02)01082-6 12464181

[13] Partida-Sánchez S Cockayne DA Monard S Jacobson EL Oppenheimer N Garvy B Cyclic ADP-ribose production by CD38 regulates intracellular calcium release, extracellular calcium influx and chemotaxis in neutrophils and is required for bacterial clearance in vivo. Nat Med (2001) 7:1209–16. 10.1038/nm1101-1209 11689885

[14] Partida-Sánchez S Iribarren P Moreno-García ME Gao J-L Murphy PM Oppenheimer N Chemotaxis and calcium responses of phagocytes to formyl peptide receptor ligands is differentially regulated by cyclic ADP ribose. J Immunol (2004) 172:1896–906. 10.4049/jimmunol.172.3.1896 14734775

[15] Partida-Sánchez S Gasser A Fliegert R Siebrands CC Dammermann W Shi G Chemotaxis of mouse bone marrow neutrophils and dendritic cells is controlled by ADP-ribose, the major product generated by the CD38 enzyme reaction. J Immunol (2007) 179:7827–39. 10.4049/jimmunol.179.11.7827 18025229

[16] Partida-Sánchez S Goodrich S Kusser K Oppenheimer N Randall TD Lund FE. Regulation of dendritic cell trafficking by the ADP-ribosyl cyclase CD38: impact on the development of humoral immunity. Immunity (2004) 20:279–91. 10.1016/S1074-7613(04)00048-2 15030772

[17] Viegas MS do Carmo A Silva T Seco F Serra V Lacerda M CD38 plays a role in effective containment of mycobacteria within granulomata and polarization of Th1 immune responses against *Mycobacterium avium*. Microbes Infect (2007) 9:847–54. 10.1016/j.micinf.2007.03.003 17533152

[18] Matalonga J Glaria E Bresque M Escande C Carbó JM Kiefer K The nuclear receptor LXR limits bacterial infection of host macrophages through a mechanism that impacts cellular NAD metabolism. Cell Rep (2017) 18:1241–55. 10.1016/j.celrep.2017.01.007 28147278

[19] Liu Z Hultin LE Cumberland WG Hultin P Schmid I Matud JL Elevated relative fluorescence intensity of CD38 antigen expression on CD8+ T cells is a marker of poor prognosis in HIV infection: results of 6 years of follow-up. Cytometry (1996) 26:1–7. 10.1002/(SICI)1097-0320(19960315)26:1<1:AID-CYTO1>3.0.CO;2-L 8809474

[20] Liu Z Cumberland WG Hultin LE Prince HE Detels R Giorgi JV. Elevated CD38 antigen expression on CD8+ T cells is a stronger marker for the risk of chronic HIV disease progression to AIDS and death in the Multicenter AIDS Cohort Study than CD4+ cell count, soluble immune activation markers, or combinations of HLA-DR and CD38 expression. J Acquir Immune Defic Syndr Hum Retrovirol (1997) 16:83–92. 9358102 10.1097/00042560-199710010-00003

[21] Giorgi JV Liu Z Hultin LE Cumberland WG Hennessey K Detels R. Elevated levels of CD38+ CD8+ T cells in HIV infection add to the prognostic value of low CD4+ T cell levels: results of 6 years of follow-up. The Los Angeles Center, Multicenter AIDS Cohort Study. J Acquir Immune Defic Syndr (1993) 6:904–12. 7686224

[22] Mocroft A Bofill M Lipman M Medina E Borthwick N Timms A CD8+,CD38+ lymphocyte percent: a useful immunological marker for monitoring HIV-1-infected patients. J Acquir Immune Defic Syndr Hum Retrovirol (1997) 14:158–62. 10.1097/00042560-199702010-00009 9052725

[23] Damle RN Wasil T Fais F Ghiotto F Valetto A Allen SL Ig V gene mutation status and CD38 expression as novel prognostic indicators in chronic lymphocytic leukemia. Blood (1999) 94:1840–7. 10477712

[24] Dürig J Naschar M Schmücker U Renzing-Köhler K Hölter T Hüttmann A CD38 expression is an important prognostic marker in chronic lymphocytic leukaemia. Leukemia (2002) 16:30–5. 10.1038/sj.leu.2402339 11840260

[25] Jiang Z Wu D Lin S Li P. CD34 and CD38 are prognostic biomarkers for acute B lymphoblastic leukemia. Biomark Res (2016) 4:23. 10.1186/s40364-016-0080-5 28018598 PMC5159997

[26] Quarona V Zaccarello G Chillemi A Brunetti E Singh VK Ferrero E CD38 and CD157: a long journey from activation markers to multifunctional molecules. Cytometry B Clin Cytom (2013) 84:207–17. 10.1002/cyto.b.21092 23576305

[27] Palumbo A Chanan-Khan A Weisel K Nooka AK Masszi T Beksac M Daratumumab, bortezomib, and dexamethasone for multiple myeloma. N Engl J Med (2016) 375:754–66. 10.1056/NEJMoa1606038 27557302

[28] Suzuki K Dimopoulos MA Takezako N Okamoto S Shinagawa A Matsumoto M Daratumumab, lenalidomide, and dexamethasone in East Asian patients with relapsed or refractory multiple myeloma: subgroup analyses of the phase 3 POLLUX study. Blood Cancer J (2018) 8:41. 10.1038/s41408-018-0071-x 29712896 PMC5928154

[29] Martin T Baz R Benson DM Lendvai N Wolf J Munster P A phase 1b study of isatuximab plus lenalidomide and dexamethasone for relapsed/refractory multiple myeloma. Blood (2017) 129:3294–303. 10.1182/blood-2016-09-740787 28483761 PMC5482100

[30] van de Donk NWCJ Janmaat ML Mutis T Lammerts van Bueren JJ Ahmadi T Sasser AK Monoclonal antibodies targeting CD38 in hematological malignancies and beyond. Immunol Rev (2016) 270:95–112. 10.1111/imr.12389 26864107 PMC4755228

[31] Kang BK Schlesinger LS. Characterization of mannose receptor-dependent phagocytosis mediated by *Mycobacterium tuberculosis* lipoarabinomannan. Infect Immun (1998) 66:2769–77. 9596746 10.1128/iai.66.6.2769-2777.1998 PMC108268

[32] Hochberg MC Updating the American College of Rheumatology revised criteria for the classification of systemic lupus erythematosus. Arthritis Rheum (1997) 40:1725 10.1002/art.1780400928 9324032

[33] Young NA Valiente GR Hampton JM Wu L-C Burd CJ Willis WL Estrogen-regulated STAT1 activation promotes TLR8 expression to facilitate signaling via microRNA-21 in systemic lupus erythematosus. Clin Immunol (2017) 176:12–22. 10.1016/j.clim.2016.12.005 28039018 PMC5815376

[34] Guerau-de-Arellano M Smith KM Godlewski J Liu Y Winger R Lawler SE Micro-RNA dysregulation in multiple sclerosis favours pro-inflammatory T-cell-mediated autoimmunity. Brain (2011) 134:3578–89. 10.1093/brain/awr262 22088562 PMC3235556

[35] Gross TJ Kremens K Powers LS Brink B Knutson T Domann FE Epigenetic silencing of the human NOS2 gene: rethinking the role of nitric oxide in human macrophage inflammatory responses. J Immunol (2014) 192:2326–38. 10.4049/jimmunol.1301758 24477906 PMC3943971

[36] Albina JE. On the expression of nitric oxide synthase by human macrophages. Why no NO? J Leukoc Biol (1995) 58:643–9. 10.1002/jlb.58.6.643 7499961

[37] Vogel DYS Glim JE Stavenuiter AWD Breur M Heijnen P Amor S Human macrophage polarization in vitro: maturation and activation methods compared. Immunobiology (2014) 219:695–703. 10.1016/j.imbio.2014.05.002 24916404

[38] Blacher E Ben Baruch B Levy A Geva N Green KD Garneau-Tsodikova S Inhibition of glioma progression by a newly discovered CD38 inhibitor. Int J Cancer (2015) 136:1422–33. 10.1002/ijc.29095 25053177

[39] Escande C Nin V Price NL Capellini V Gomes AP Barbosa MT Flavonoid apigenin is an inhibitor of the NAD+ ase CD38: implications for cellular NAD+ metabolism, protein acetylation, and treatment of metabolic syndrome. Diabetes (2013) 62:1084–93. 10.2337/db12-1139 23172919 PMC3609577

[40] Sun H Luo G Chen D Xiang Z. A comprehensive and system review for the pharmacological mechanism of action of rhein, an active anthraquinone ingredient. Front Pharmacol (2016) 7:247. 10.3389/fphar.2016.00247 27582705 PMC4987408

[41] Mead JR Hughes TR Irvine SA Singh NN Ramji DP. Interferon-gamma stimulates the expression of the inducible cAMP early repressor in macrophages through the activation of casein kinase 2. A potentially novel pathway for interferon-gamma-mediated inhibition of gene transcription. J Biol Chem (2003) 278:17741–51. 10.1074/jbc.M301602200 12609974

[42] Bombardier C Gladman DD Urowitz MB Caron D Chang CH. Derivation of the SLEDAI. A disease activity index for lupus patients. The Committee on Prognosis Studies in SLE. Arthritis Rheum (1992) 35:630–40. 10.1002/art.1780350606 1599520

[43] Ziegler-Heitbrock L Ancuta P Crowe S Dalod M Grau V Hart DN Nomenclature of monocytes and dendritic cells in blood. Blood (2010) 116:e74–80. 10.1182/blood-2010-02-258558 20628149

[44] Ziegler-Heitbrock L. Blood monocytes and their subsets: established features and open questions. Front Immunol (2015) 6:423. 10.3389/fimmu.2015.00423 26347746 PMC4538304

[45] Zhao C Zhang H Wong W-C Sem X Han H Ong SM Identification of novel functional differences in monocyte subsets using proteomic and transcriptomic methods. J Proteome Res (2009) 8:4028–38. 10.1021/pr900364p 19514703

[46] Mobley JL Leininger M Madore S Baginski TJ Renkiewicz R. Genetic evidence of a functional monocyte dichotomy. Inflammation (2007) 30:189–97. 10.1007/s10753-007-9036-0 17587162

[47] Ingersoll MA Spanbroek R Lottaz C Gautier EL Frankenberger M Hoffmann R Comparison of gene expression profiles between human and mouse monocyte subsets. Blood (2010) 115:e10–9. 10.1182/blood-2009-07-235028 19965649 PMC2810986

[48] Cros J Cagnard N Woollard K Patey N Zhang S-Y Senechal B Human CD14dim monocytes patrol and sense nucleic acids and viruses via TLR7 and TLR8 receptors. Immunity (2010) 33:375–86. 10.1016/j.immuni.2010.08.012 20832340 PMC3063338

[49] Ancuta P Liu K-Y Misra V Wacleche VS Gosselin A Zhou X Transcriptional profiling reveals developmental relationship and distinct biological functions of CD16+ and CD16- monocyte subsets. BMC Genomics (2009) 10:403. 10.1186/1471-2164-10-403 19712453 PMC2741492

[50] Wong KL Tai JJ-Y Wong W-C Han H Sem X Yeap W-H Gene expression profiling reveals the defining features of the classical, intermediate, and nonclassical human monocyte subsets. Blood (2011) 118:e16–31. 10.1182/blood-2010-12-326355 21653326

[51] Zawada AM Rogacev KS Rotter B Winter P Marell R-R Fliser D SuperSAGE evidence for CD14++CD16+ monocytes as a third monocyte subset. Blood (2011) 118:e50–61. 10.1182/blood-2011-01-326827 21803849

[52] Frankenberger M Sternsdorf T Pechumer H Pforte A Ziegler-Heitbrock HW. Differential cytokine expression in human blood monocyte subpopulations: a polymerase chain reaction analysis. Blood (1996) 87:373–7. 8547664

[53] Belge K-U Dayyani F Horelt A Siedlar M Frankenberger M Frankenberger B The proinflammatory CD14+CD16+DR++ monocytes are a major source of TNF. J Immunol (2002) 168:3536–42. 10.4049/jimmunol.168.7.3536 11907116

[54] Mukherjee R Kanti Barman P Kumar Thatoi P Tripathy R Kumar Das B Ravindran B. Non-classical monocytes display inflammatory features: validation in sepsis and systemic lupus erythematous. Sci Rep (2015) 5:13886. 10.1038/srep13886 26358827 PMC4566081

[55] Thaler B Hohensinner PJ Krychtiuk KA Matzneller P Koller L Brekalo M Differential in vivo activation of monocyte subsets during low-grade inflammation through experimental endotoxemia in humans. Sci Rep (2016) 6:30162. 10.1038/srep30162 27444882 PMC4957086

[56] Musso T Deaglio S Franco L Calosso L Badolato R Garbarino G CD38 expression and functional activities are up-regulated by IFN-gamma on human monocytes and monocytic cell lines. J Leukoc Biol (2001) 69:605–12. 10.1189/jlb.69.4.605 11310847

[57] Kang B-N Tirumurugaan KG Deshpande DA Amrani Y Panettieri RA Walseth TF Transcriptional regulation of CD38 expression by tumor necrosis factor-alpha in human airway smooth muscle cells: role of NF-kappaB and sensitivity to glucocorticoids. FASEB J (2006) 20:1000–2. 10.1096/fj.05-4585fje 16571778

[58] Saborit-Villarroya I Vaisitti T Rossi D D’Arena G Gaidano G Malavasi F E2A is a transcriptional regulator of CD38 expression in chronic lymphocytic leukemia. Leukemia (2011) 25:479–88. 10.1038/leu.2010.291 21212793

[59] Lund FE. Signaling properties of CD38 in the mouse immune system: enzyme-dependent and -independent roles in immunity. Mol Med (2006) 12:328–33. 10.2119/2006-00099.Lund 17380200 PMC1829203

[60] Hu F Xing F Zhu G Xu G Li C Qu J Rhein antagonizes P2X7 receptor in rat peritoneal macrophages. Sci Rep (2015) 5:14012. 10.1038/srep14012 26354875 PMC4564849

[61] Gao Y Chen X Fang L Liu F Cai R Peng C Rhein exerts pro- and anti-inflammatory actions by targeting IKKβ inhibition in LPS-activated macrophages. Free Radic Biol Med (2014) 72:104–12. 10.1016/j.freeradbiomed.2014.04.001 24721152

[62] Zhang X Wang G Gurley EC Zhou H. Flavonoid apigenin inhibits lipopolysaccharide-induced inflammatory response through multiple mechanisms in macrophages. PLoS One (2014) 9:e107072. 10.1371/journal.pone.0107072 25192391 PMC4156420

[63] Arango D Diosa-Toro M Rojas-Hernandez LS Cooperstone JL Schwartz SJ Mo X Dietary apigenin reduces LPS-induced expression of miR-155 restoring immune balance during inflammation. Mol Nutr Food Res (2015) 59:763–72. 10.1002/mnfr.201400705 25641956 PMC7955240

[64] Jablonski KA Gaudet AD Amici SA Popovich PG Guerau-de-Arellano M. Control of the inflammatory macrophage transcriptional signature by miR-155. PLoS One (2016) 11:e0159724. 10.1371/journal.pone.0159724 27447824 PMC4957803

[65] Feng X Weng D Zhou F Owen YD Qin H Zhao J Activation of PPARγ by a natural flavonoid modulator, apigenin ameliorates obesity-related inflammation via regulation of macrophage polarization. EBioMedicine (2016) 9:61–76. 10.1016/j.ebiom.2016.06.017 27374313 PMC4972579

[66] Partida-Sánchez S Randall TD Lund FE. Innate immunity is regulated by CD38, an ecto-enzyme with ADP-ribosyl cyclase activity. Microbes Infect (2003) 5:49–58. 10.1016/S1286-4579(02)00055-2 12593973

[67] Höpken UE Lipp M. All roads lead to Rome: triggering dendritic cell migration. Immunity (2004) 20:244–6. 10.1016/S1074-7613(04)00056-1 15030768

[68] Becker AM Dao KH Han BK Kornu R Lakhanpal S Mobley AB SLE peripheral blood B cell, T cell and myeloid cell transcriptomes display unique profiles and each subset contributes to the interferon signature. PLoS One (2013) 8:e67003. 10.1371/journal.pone.0067003 23826184 PMC3691135

[69] Pavón EJ Zumaquero E Rosal-Vela A Khoo K-M Cerezo-Wallis D García-Rodríguez S Increased CD38 expression in T cells and circulating anti-CD38 IgG autoantibodies differentially correlate with distinct cytokine profiles and disease activity in systemic lupus erythematosus patients. Cytokine (2013) 62:232–43. 10.1016/j.cyto.2013.02.023 23538292

[70] Henriques A Silva I Inês L Souto-Carneiro MM Pais ML Trindade H CD38, CD81 and BAFFR combined expression by transitional B cells distinguishes active from inactive systemic lupus erythematosus. Clin Exp Med (2016) 16:227–32. 10.1007/s10238-015-0348-3 25894569

[71] Vidal S Kono DH Theofilopoulos AN. Loci predisposing to autoimmunity in MRL-Fas lpr and C57BL/6-Faslpr mice. J Clin Invest (1998) 101:696–702. 10.1172/JCI1817 9449705 PMC508615

[72] González-Escribano MF Aguilar F Torres B Sánchez-Román J Núñez-Roldán A. CD38 polymorphisms in Spanish patients with systemic lupus erythematosus. Hum Immunol (2004) 65:660–4. 10.1016/j.humimm.2004.02.032 15219386

[73] Hiepe F Radbruch A. Plasma cells as an innovative target in autoimmune disease with renal manifestations. Nat Rev Nephrol (2016) 12:232–40. 10.1038/nrneph.2016.20 26923204

[74] Center for Drug Evaluation and Research Application Number 761036Origs000. Center for Drug Evaluation and Research Application Number 761036Origs000. Available from: https://www.accessdata.fda.gov/drugsatfda_docs/nda/2015/761036Orig1s000PharmR.pdf (Accessed: February 11, 2017).

[75] Postigo J Iglesias M Cerezo-Wallis D Rosal-Vela A García-Rodríguez S Zubiaur M Mice deficient in CD38 develop an attenuated form of collagen type II-induced arthritis. PLoS One (2012) 7:e33534. 10.1371/journal.pone.0033534 22438945 PMC3306406

[76] Viegas MS Silva T Monteiro MM do Carmo A Martins TC. Knocking out of CD38 accelerates development of a lupus-like disease in lpr mice. Rheumatology (Oxford) (2011) 50:1569–77. 10.1093/rheumatology/ker178 21586522

[77] Chen J Chen Y-G Reifsnyder PC Schott WH Lee C-H Osborne M Targeted disruption of CD38 accelerates autoimmune diabetes in NOD/Lt mice by enhancing autoimmunity in an ADP-ribosyltransferase 2-dependent fashion. J Immunol (2006) 176:4590–9. 10.4049/jimmunol.176.8.4590 16585549

