# Supplementary figures and images for: CD38 Is Robustly Induced in Human Macrophages and Monocytes in Inflammatory Conditions
Source: Front Immunol. 2018 Jul 10;9:1593. doi: 10.3389/fimmu.2018.01593 (PMC6048227; doi:10.3389/fimmu.2018.01593)

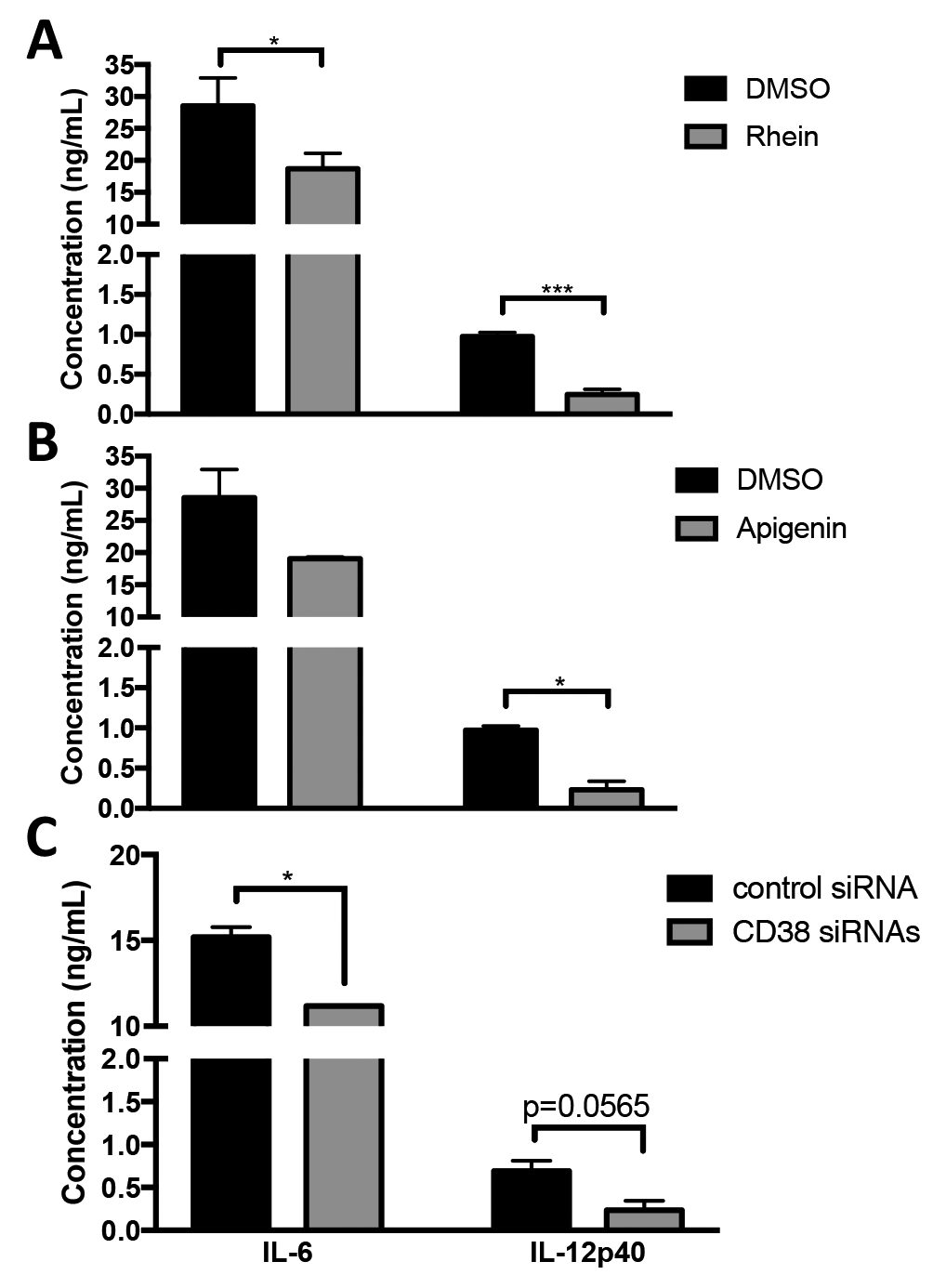

Supplement: Figure S1 — Representative graphs with absolute values from individual experiments for Figure 3. Human monocyte-derived macrophages (MDMs) were treated with 15 µM rhein or DMSO control (A), 25 µM apigenin or DMSO (B), or transfected with 100 µM CD38 siRNA cocktail or nonsense siRNA control (C) on day 5. On day 6, they were activated with LPS + IFN-γ for an additional 24 h prior to analysis. (A–C) IL-6 and IL-12p40 secretion was analyzed by ELISA from MDM supernatants. Graphs show data (relative to corresponding experiment vehicle control or nonsense siRNA) from a single experiment, one to two biological replicates with two technical replicates per sample. All data were analyzed by unpaired t tests. *p < 0.05, **p < 0.01, ***p < 0.001. [file image_1.TIF]

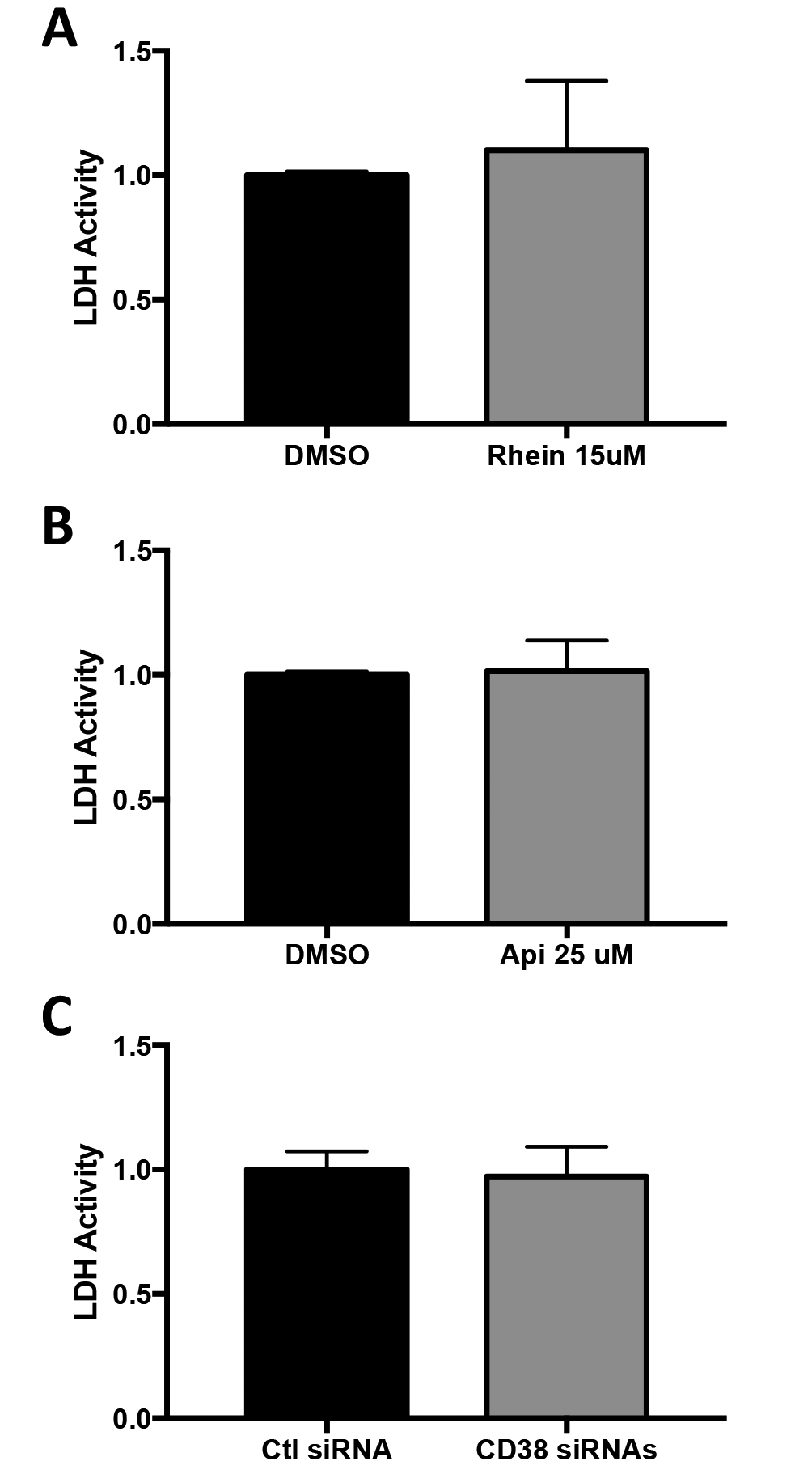

Supplement: Figure S2 — Lactate dehydrogenase (LDH) remains unchanged in treatment groups compared with corresponding experiment vehicle control or nonsense siRNA. (A–C) Quantification of LDH activity from monocyte-derived macrophage supernatants of 15 µM rhein-treated (A), 25 µM apigenin-treated (B), or CD38 siRNA-transfected (C) conditions compared with control conditions shown in Figure 3. All graphs represent pooled data from three to five independent experiments with one to two biological replicates per experiment and two to three technical replicates per sample. All data were analyzed by unpaired t tests. [file image_2.TIF]

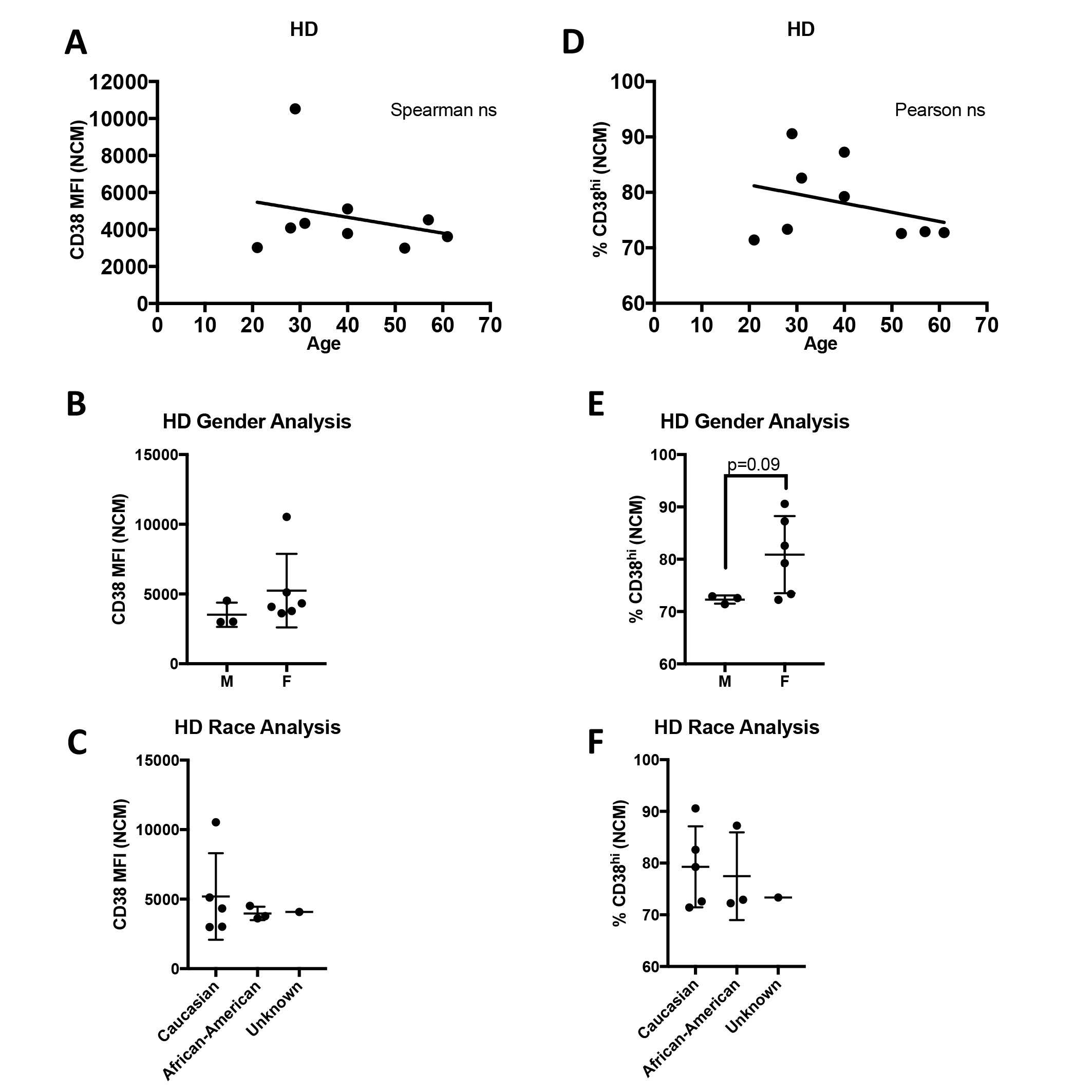

Supplement: Figure S3 — There are no significant associations between CD38 expression and age, gender, or race. CD38 mean fluorescence intensity (MFI) (A–C) and % CD38hi cells (D–F) from healthy donors (HD) were analyzed to determine if age, gender or race affected CD38 protein load or surface expression. (A,D) Correlation analyses of SLE Disease Activity Index score with non-classical monocytes (NCM) CD38 MFI (A) or % CD38hi NCM (D) in HD. (A) CD38 MFI did not pass D’Agostino and Pearson normality test, so Spearman correlation analysis was performed, r = −0.1506, p = ns, n = 9. (D) Percent of CD38hi NCM has Gaussian distribution as determined by D’Agostino and Pearson normality test, so Pearson correlation analysis was performed, r = −0.2617, p = ns, n = 9. (B,C,E,F) CD38 MFI (B,C) and % CD38hi cells (E,F) in NCM from HD were analyzed by gender (B,D), n = 3 males (M) and 6 females (F); or race (C,F), n = 5 Caucasians; n = 3 African-Americans, n = 1 unknown. Data analyzed by unpaired t tests, p = ns. [file image_3.TIF]

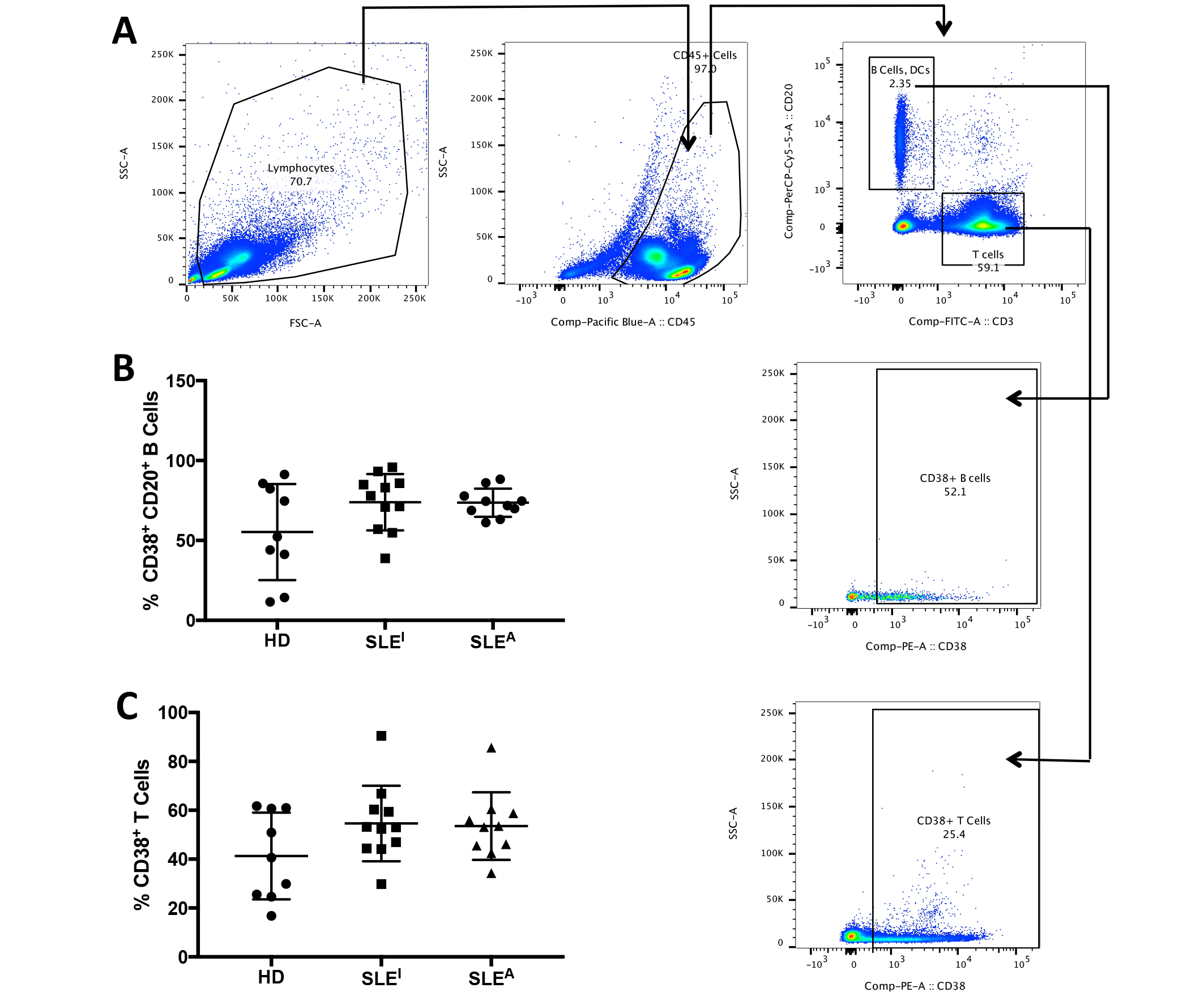

Supplement: Figure S4 — CD38+ T cell and B cell percentages in patients with SLE. (A) Gating strategy: peripheral blood mononuclear cells isolated from healthy donors or SLE patients (panel 1) were gated for CD45+ cells (panel 2), then CD20+ or CD3+ cells (panel 3), and subsequently CD38+ cells (B,C). (B,C) Quantification of CD45+CD20+CD38+ (B) or CD45+CD3+CD38+ (C) cells are expressed as percent of positive cells ± SD, n = 9 for HD, n = 11 for SLEI, n = 10 for SLEA. One-way analysis of variance with Tukey’s multiple comparisons test. [file image_4.TIF]
